# Optimizing antibody affinity and stability by the automated design of the variable light-heavy chain interfaces

**DOI:** 10.1371/journal.pcbi.1007207

**Published:** 2019-08-23

**Authors:** Shira Warszawski, Aliza Borenstein Katz, Rosalie Lipsh, Lev Khmelnitsky, Gili Ben Nissan, Gabriel Javitt, Orly Dym, Tamar Unger, Orli Knop, Shira Albeck, Ron Diskin, Deborah Fass, Michal Sharon, Sarel J. Fleishman

**Affiliations:** 1 Department of Biomolecular Sciences, Weizmann Institute of Science, Rehovot, Israel; 2 Department of Structural Biology, Weizmann Institute of Science, Rehovot, Israel; 3 Israel Structural Proteomics Center, Weizmann Institute of Science, Rehovot, Israel; Koç University, TURKEY

## Abstract

Antibodies developed for research and clinical applications may exhibit suboptimal stability, expressibility, or affinity. Existing optimization strategies focus on surface mutations, whereas natural affinity maturation also introduces mutations in the antibody core, simultaneously improving stability and affinity. To systematically map the mutational tolerance of an antibody variable fragment (Fv), we performed yeast display and applied deep mutational scanning to an anti-lysozyme antibody and found that many of the affinity-enhancing mutations clustered at the variable light-heavy chain interface, within the antibody core. Rosetta design combined enhancing mutations, yielding a variant with tenfold higher affinity and substantially improved stability. To make this approach broadly accessible, we developed AbLIFT, an automated web server that designs multipoint core mutations to improve contacts between specific Fv light and heavy chains (http://AbLIFT.weizmann.ac.il). We applied AbLIFT to two unrelated antibodies targeting the human antigens VEGF and QSOX1. Strikingly, the designs improved stability, affinity, and expression yields. The results provide proof-of-principle for bypassing laborious cycles of antibody engineering through automated computational affinity and stability design.

## Author summary

Antibodies are highly important in research, biotechnology, and medical applications. Despite their great utility, however, many antibodies exhibit suboptimal stability and affinity, raising production costs and limiting their practical usefulness. To tackle this general limitation, we used deep mutational scanning to characterize the effects of mutations in an antibody variable fragment on its antigen-binding affinity. Surprisingly, many of the affinity-enhancing mutations clustered at the variable light-heavy chain interface. We, therefore, developed an automated method, called AbLIFT (http://AbLIFT.weizmann.ac.il) to optimize this interface through design. Two unrelated antibodies were tested and showed improvements in expression levels, stability, and antigen-binding affinity. Since AbLIFT requires testing of only a few dozen specific designs, it may dramatically accelerate the development of promising antibodies into useful research and clinical tools.

## Introduction

High-affinity natural antibodies are generated through an iterative process of mutation and selection for antigen binding known as affinity maturation. Affinity maturation also selects antibodies that exhibit higher stability and expressibility [1], both of which are essential parameters in the development of antibodies into research or medical tools [2]. In recent decades, synthetic antibody repertoires have been widely adopted in antibody discovery and optimization, providing greater control over the selection process than animal immunization. In this approach, a library of antibody variable fragments (Fv) is displayed, for instance on yeast cells, and screened to select high-affinity binders or to improve the affinity of existing antibodies [3]. These methods are powerful [4,5], but a large fraction of high-affinity antibodies isolated from synthetic repertoires exhibits impaired stability [6]. Impaired stability can limit expression yields and increase aggregation propensity [7], resulting in high production costs [8], fast antibody clearance from circulation and adverse immune responses in patients [9]. Thus, the tradeoff between antibody stability (including solubility and expressibility) and affinity can delay and even block the development of antibodies in research and medicine [10]. General methods to improve antibody stability while maintaining or even increasing affinity are therefore urgently needed to reduce the attrition rate in antibody development pipelines [11].

To boost antibody stability and affinity, computational design methods have been developed. These have focused on the Fv complementarity-determining regions (CDRs), which are typically in direct contact with the antigen. Some methods, for instance, improved electrostatic complementarity with the antigen [12] or eliminated hydrophobic surface patches [13–18]. Natural and laboratory affinity maturation, by contrast, introduce mutations in both the CDRs and the antibody core [1,5]. Core mutations may improve antibody stability by eliminating packing defects, and they may enhance affinity by preorganizing the antigen-binding site [1]. Although mutations in the core may contribute less to affinity than ones in the CDRs, they are more likely to retain the intricate structure of the antigen-binding site and are therefore likely to be compatible with affinity-enhancing mutations in the CDRs that were obtained through other optimization strategies. The antibody core, however, is a large and densely packed region, complicating the design of improved variants [5,19]. For instance, we recently presented and validated an automated computational strategy, called PROSS [20], for protein-stability design. Similar to other stability design algorithms [21], however, PROSS only rarely introduces core mutations and does not improve binding affinity [22]. Reliable prediction of mutational effects in the antibody core and especially successful design of networks of interacting multipoint core mutations has, therefore, remained a challenge [21,23].

Recently, deep mutational scanning has been successfully applied to study the mutational tolerance of antibodies and other binders [24–30]. In this approach, amino acid positions in the binder are systematically mutated to all of the natural amino acid identities; the mutants are pooled into one library containing all single-point mutations; populations of binders are selected from this library using *in vitro* display and high-throughput screening; and the selected and unselected populations are subjected to deep-sequencing analysis to infer which mutations are enriched relative to the starting binder, thus systematically identifying affinity-enhancing mutations. Deep mutational scanning has been very successfully used to guide protein design and engineering of improved binders [24,26,31,32] but has not yet been exploited to improve protein-design methodology itself. The large improvements in the reliability and breadth of detection of affinity-enhancing mutations through deep mutational scanning inspired us to revisit the challenge of accurately predicting the effects of core mutations on antibody affinity and stability. Deep mutational scanning guided us in uncovering a cluster of core positions at the light-heavy chain (vL-vH) interface where many affinity-enhancing mutations occurred. We then used these systematic data to establish general rules for computational design of antibody Fvs with improved vL-vH interactions; we implemented these rules as an automated method, called AbLIFT and made it available through a web server (http://AbLIFT.weizmann.ac.il). AbLIFT designs exhibited striking gains in affinity, stability, and expressibility in two unrelated antibodies that target the human disease markers Vascular-Epidermal Growth Factor (VEGF) and the enzyme Quiescin Sulfhydryl Oxidase 1 (QSOX1).

## Results

### A cluster of affinity-enhancing mutations at the vL-vH interface

**Fig 1:**
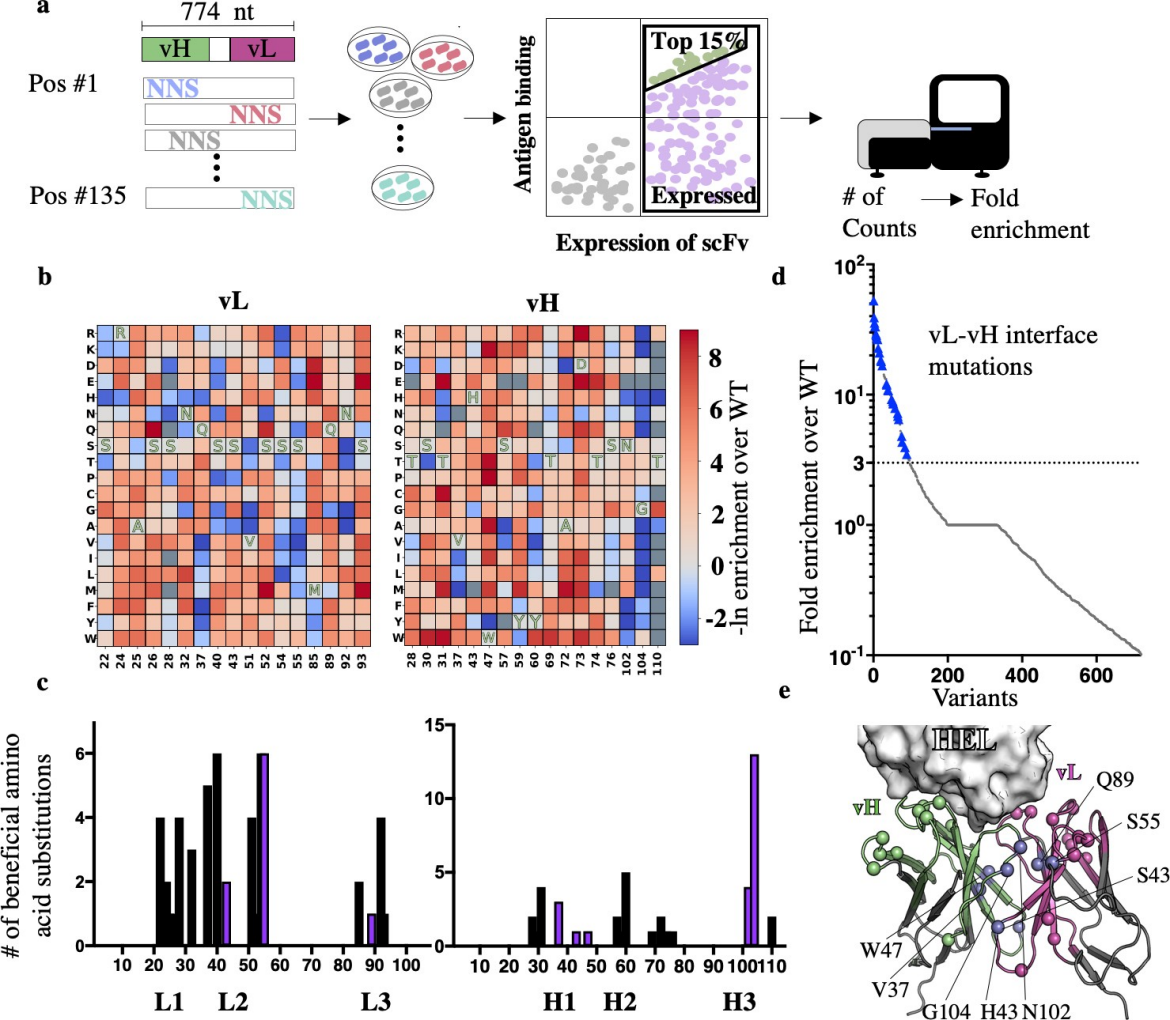
Deep mutational scanning of an antibody variable fragment (Fv). (**a**) 135 positions across the Fv of the anti-lysozyme antibody D44.1 (encompassing the vL-vH interface and all positions from the antigen-binding surface to the level of the disulfide-linked cysteines) were individually diversified using degenerate codons (NNS) to encode all point mutations. The variants were transformed into yeast cells, subjected to low-stringency selection for binding (green spots) and for expression (purple + green spots), and then to deep-sequencing analysis. (**b**) 34 Fv positions exhibited enhanced binding upon mutation (blue). Amino acids (x-axis) are numbered according to ref. [33]. Blue, red and gray encode mutations that are enriched, depleted, or ones with insufficient data, respectively. Amino acid identities in the parental antibody are indicated in one-letter code for each position. (**c**) At several positions, more than five alternative identities enhanced affinity, indicating that the combinatorial sequence space of affinity-enhancing multipoint mutants is large. Positions at the vL-vH interface are colored purple. (**d**) Mutations were ranked according to their enrichment ratios, revealing that many (30%) of the top affinity-enhancing mutations occurred at the vL-vH interface (30 identities at the eight positions with enrichment over the parental identity above threefold are marked in blue triangles). (**e**) Spheres indicate positions on the molecular structure of the bound HEL-D44.1 complex (PDB entry 1MLC) in which mutations exhibited at least threefold enrichment relative to D44.1. A cluster of enriched positions at the vL-vH interface, mostly belonging to the antibody framework, is highlighted in purple. HEL—hen egg-white lysozyme.

To study the mutational tolerance of an antibody Fv, we selected 135 positions in the anti-lysozyme antibody D44.1 [33] for deep mutational scanning (**S1A Fig**). The positions encompassed most of the CDRs, the vL-vH interface and additional peripheral positions (**Fig 1A**). D44.1 and each point mutant were genetically encoded as single-chain variable fragments (scFv) in which the heavy chain was fused to the light chain via a flexible linker, and the genes were transformed into yeast cells for binding and expression screens by yeast display [3]. Following incubation with hen egg-white lysozyme, the top 15% binders were selected from this library, and the same library was also subjected to low-stringency selection for expression levels to provide a baseline. The plasmids containing scFv-encoding genes from both selections were purified, amplified by PCR, and subjected to deep sequencing, resulting in 8 million high-quality reads [32]. We then determined the enrichment of each mutant relative to D44.1 as the ratio between populations selected for binding and expression (**Fig 1B**).

We found affinity-enhancing mutations at 34 positions, mostly within the CDRs, as expected (**Fig 1C**). We also noticed a surprisingly large cluster of eight positions at the vL-vH interface where affinity-enhancing mutations occurred, although they were not in direct contact with the antigen (**Fig 1D and 1E**). This cluster of affinity-enhancing mutations in the vL-vH interface is intriguing for four reasons: (1) the vL-vH interface mediates the assembly of the Fv from the two antibody chains, and mutations in this region have the potential to also enhance stability through improved Fv assembly [1]; (2) the genetic pairing of light and heavy chains during germline antibody generation is a random process which may result in suboptimal vL-vH interfaces, flexibility in the antigen-binding site [34], and therefore in lower antigen affinity [35]; (3) this region is distant from the mutational hotspots in the CDRs and may not be fully optimized in the course of natural affinity maturation [36]; and (4) antibody-engineering procedures, such as humanization or CDR grafting may inadvertently compromise the structural integrity of this region by mispairing CDRs and frameworks [37]. Based on these considerations, we hypothesized that the vL-vH interface may be especially amenable to the design of multipoint mutants that simultaneously improve stability and affinity in both natural and engineered antibodies.

Combining mutations in densely packed protein cores, such as the vL-vH interface, is challenging, however, because inadvertently introduced voids, steric overlaps, or mispaired polar amino acid side-chains can lead to protein instability, misfolding, and aggregation [21,23]. We, therefore, asked whether the mutational-tolerance map could guide Rosetta design in finding improved multipoint mutants at the vL-vH interface. In preliminary calculations starting from the lysozyme-bound D44.1 structure (PDB entry: 1MLC), we restricted Rosetta combinatorial design to the eight positions and 38 identities (including the wild type identities) at these positions that showed at least threefold enrichment relative to D44.1 according to the mutational-tolerance map (**Fig 1B and 1E**). The resulting design, however, comprised only three conservative mutations, suggesting that the dense packing and backbone rigidity at the vL-vH interface restricted sequence optimization. We, therefore, repeated design calculations but this time excluded the wild type identities at the eight positions, forcing the design of an optimal combination of mutations only from those that were substantially enriched in deep mutational scanning. We iterated sequence design and backbone and sidechain minimization to promote the acceptance of even radical mutations yielding design D44.1^des^ with eight mutations. As a preliminary qualitative test, we analyzed D44.1^des^ binding to lysozyme and to seven of the eight single-point mutations formatted as scFvs using yeast display [3]. As expected, each of the point mutations improved the apparent binding affinity relative to D44.1; and yet, the multipoint D44.1^des^ exhibited a substantial improvement in apparent affinity compared to the single-point mutations (**S1B Fig**).

**Fig 2:**
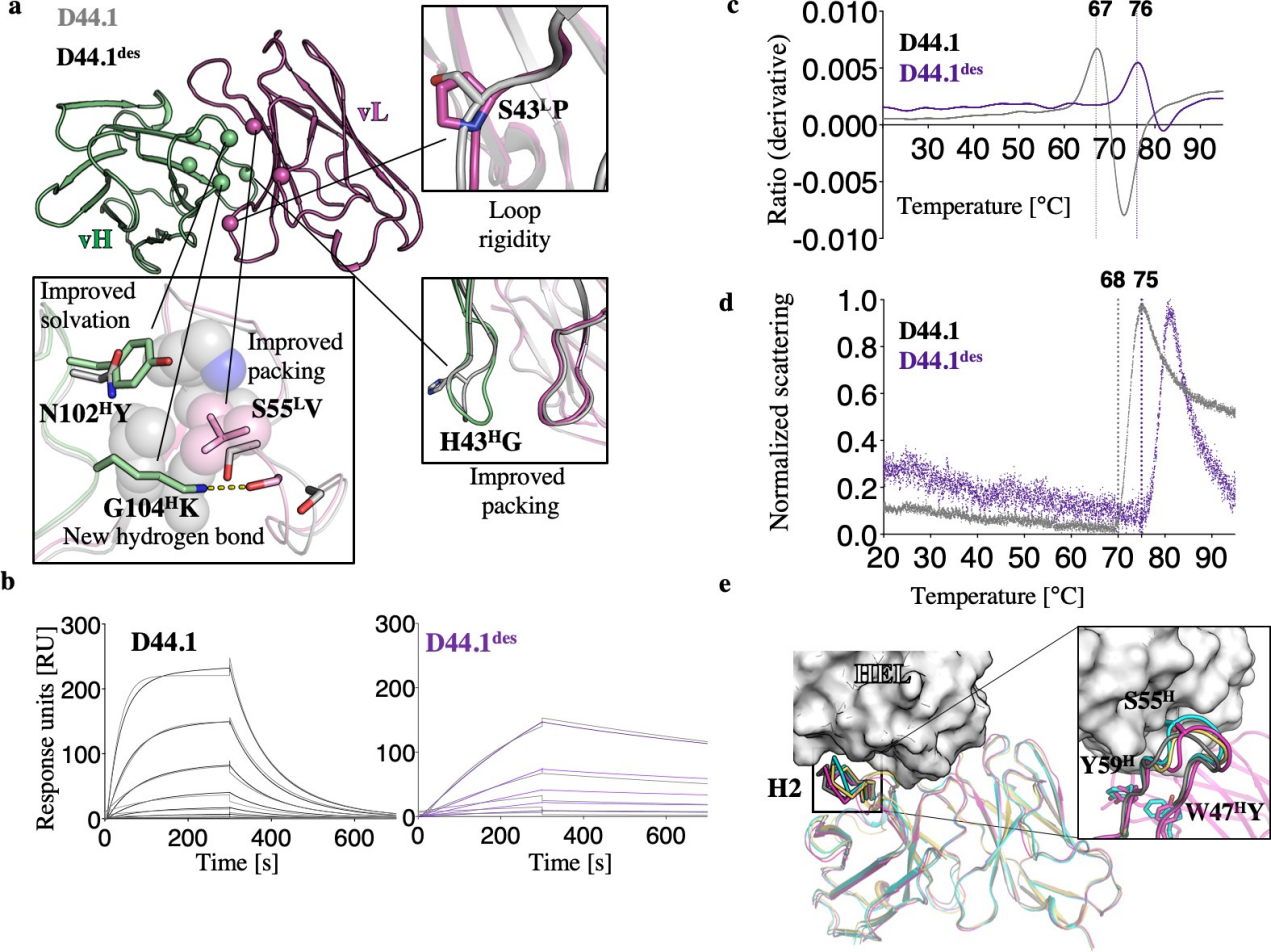
Gains in affinity, stability, and aggregation resistance through vL-vH interface design guided by deep mutational scanning. (**a**) Comparison of the starting anti-lysozyme antibody D44.1 and the crystal structure of design D44.1^des^ (PDB entries: 1MLC and 6GC2, respectively) showed improved interactions across the interface and likely increased backbone rigidity. (**b**) SPR kinetic analysis of hen egg-white lysozyme (HEL) binding with threefold dilutions of HEL from a maximal concentration of 333 nM for D44.1 and 111 nM for D44.1^des^ (kinetic fits shown in gray). D44.1 exhibited *k*_*a*_ = 1.5 * 10^5^ M^-1^s^-1^, *k*_*d*_ = 0.021 s^-1^, and *K*_*D*_ = 137 nM. D44.1^des^ exhibited *k*_*a*_ = 5.3 * 10^4^ M^-1^s^-1^, *k*_*d*_ = 7.9 * 10^−4^ s^-1^, and *K*_*D*_ = 15 nM (**c & d**) Thermal denaturation and temperature of aggregation onset, respectively, of D44.1 and D44.1^des^ formatted as Fabs using microscale thermophoresis. (**e**) A potential molecular explanation for improved affinity. The unbound (cyan) and bound (gray) structures of D44.1 (PDB entries: 1MLB and 1MLC, respectively) exhibit a different H2 backbone conformation; the former sterically hinders lysozyme binding. The high-affinity anti-lysozyme antibody F10.6.6 in its unbound form (PDB entry: 2Q76; orange) and D44.1^des^ (pink) are similar to one another and to the bound conformation of D44.1 and are compatible with binding HEL. (inset) a closeup of the H2 backbone conformation revealing that the D44.1 H2 backbone (cyan) sterically overlaps with lysozyme, whereas all the other backbone conformations are compatible with lysozyme binding. The Trp47^H^Tyr mutation in D44.1^des^ alters packing at the base of CDR H2 and may induce the observed conformational change in the design.

To determine what molecular factors might underlie higher affinity in D44.1^des^, we expressed the design as an antigen-binding fragment (Fab) and determined its structure by X-ray crystallography in the absence of lysozyme (**S1 Table**). Despite eight core mutations, the overall agreement between D44.1^des^ and the bound structure of D44.1, which served as the starting point for designing D44.1^des^, was excellent (**S2 Fig**): The two structures deviated by <1 Å backbone root-mean-square deviation (rmsd) and in side-chain residues comprising the lysozyme-binding site. The mutations apparently improved various molecular aspects of the vL-vH interface including interface packing, solvation, and backbone rigidity (**Fig 2A**). Next, we tested lysozyme binding by D44.1 and D44.1^des^ (both expressed and purified as Fab) using surface-plasmon resonance (SPR). D44.1^des^ exhibited nearly tenfold improvement in affinity (*K*_*D*_ of 15 versus 135 nM for D44.1^des^ and D44.1, respectively), with a 25-fold slower off-rate (8 * 10^−4^ s^-1^) (**Fig 2B**). D44.1^des^ also exhibited improved thermal denaturation and aggregation resistance (**Fig 2C and 2D**).

We also compared the molecular structure of D44.1^des^ to the unbound structure of D44.1 (PDB entry: 1MLB). The main difference between the two structures was localized to the backbone conformation of CDR H2: Whereas H2 in the unbound structure of D44.1 adopts a conformation that would sterically overlap with lysozyme in the bound structure, the H2 backbone of D44.1^des^ moves away from this position such that, even in the unbound state, the design is sterically compatible with lysozyme binding. The H2 backbone conformation of D44.1^des^ is not identical but is similar to the H2 conformation in the bound D44.1 structure and also to the conformation observed in the unbound structure of the high-affinity anti-lysozyme antibody F10.6.6 (PDB entry: 1P2C) (**Fig 2E**). Although it is possible that the observed conformational differences among the structures are due to differences in crystallographic conditions, we note that the mutation Trp47^H^Tyr in D44.1^des^ is incompatible with the observed H2 conformation in the unbound state of D44.1 and may induce the observed change in the design’s backbone conformation. Hence, the structure-based analysis suggested that the design of the vL-vH interface based on the bound antibody structure might increase the compatibility of the CDR backbones for the ligand while simultaneously improving stability.

### Computational mutation-tolerance mapping

The successful optimization of antibody affinity and stability encouraged us to fully automate the design procedure, eliminating the requirement for experimental deep mutational scanning. We, therefore, sought a general computational strategy that would predict which mutations in the vL-vH interface were likely to enhance affinity and stability, with the goal of developing a general computational procedure for mutational-tolerance mapping. To achieve this goal, we exploited the large experimental dataset of the D44.1 mutational tolerance map, comprising 2,294 point mutations, for training. At each of the mutated D44.1 Fv positions, we used Rosetta to compute the changes to native-state energy due to each of the 19 amino acid mutations (ΔΔ*G*). Using a multiple-sequence alignment of homologous Fvs, we additionally computed each point mutation’s evolutionary-conservation score, as represented in a Position-Specific Scoring Matrix (PSSM) [38]. These two computed parameters provide complementary predictions of mutational tolerance: the former predicts the impact of a mutation on native-state stability and the latter discriminates between evolutionarily tolerated mutations and those that have been purged by evolution. The use of these two parameters has recently led to substantial improvement in design accuracy in binder and enzyme design challenges in our laboratory [20–22,38–43]. We specifically used these two parameters because they can be computed for any antibody given an accurate experimental or model structure, allowing us, in principle, to compute mutational tolerance maps for any antibody Fv.

**Fig 3:**
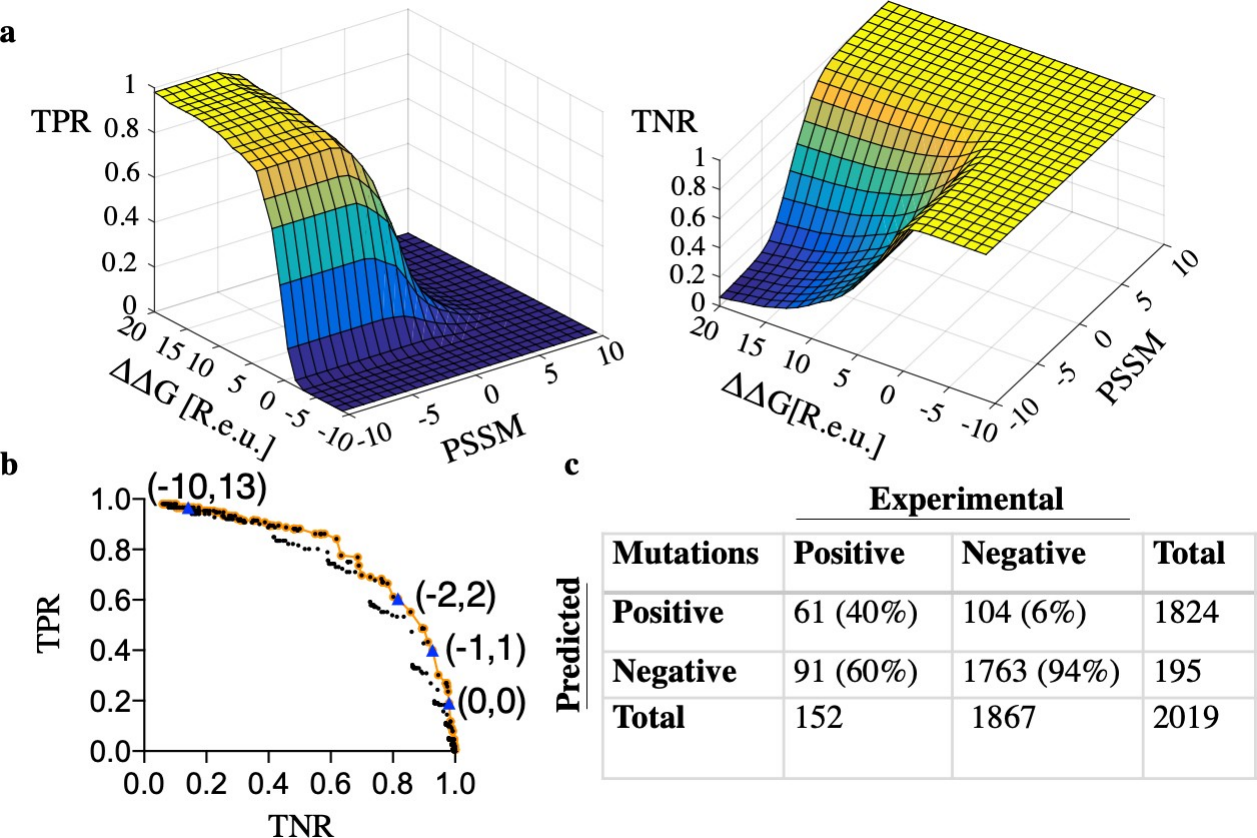
Mutational-tolerance mapping by Rosetta atomistic energy calculations (ΔΔ*G*) and evolutionary-conservation scores (PSSM). (**a**) Systematic analysis of combinations of PSSM and ΔΔ*G* thresholds reveals an expected tradeoff in prediction accuracy of mutational tolerance. Each combination of thresholds (-10≤PSSM≤10; -10≤ΔΔ*G*≤20 Rosetta energy units, R.e.u.) results in a different fraction of correctly predicted enhancing or deleterious mutations (true-positive rate [TPR] and true-negative rate [TNR], respectively) observed in the deep mutational scanning data of D44.1. (**b**) All (PSSM, ΔΔ*G*) combinations are plotted with their TPR and TNR values, and the Pareto-optimal front is indicated in orange. Several combinations of (PSSM, ΔΔ*G*) thresholds are indicated by blue triangles. (**c**) The thresholds (PSSM≥-1, ΔΔ*G*≤+1 R.e.u.) result in a TNR of 94% and TPR of 40% and were used in subsequent design calculations. Optimal ΔΔ*G* cutoffs may vary depending on the energy function and the relaxation protocol. For details on these choices, see Methods.

We systematically screened different combinations of ΔΔ*G* and PSSM thresholds to determine which combination optimally discriminates enhancing from deleterious mutations as observed in the experimental mutational-tolerance map of D44.1. We defined the prediction true-positive rate (TPR) as the proportion of correctly predicted affinity-enhancing mutations (>1.5-fold enrichment according to deep mutational scanning) and the true-negative rate (TNR) as the proportion of correctly predicted deleterious ones (enrichment ratio <1). The resulting phase space of (PSSM, ΔΔ*G*) thresholds revealed an expected tradeoff, wherein high TNR came at the cost of low TPR, and *vice versa* (**Fig 3A**). The likelihood of obtaining a multipoint mutant without a single deleterious mutation can be roughly approximated by TNR^*n*^, where *n* is the number of mutations. Given the large size of the vL-vH interface (20–30 positions [44]), we aimed for a large maximum number of mutations in each multipoint mutant (*n* = 10) and therefore selected a stringent cutoff TNR = 94%, providing a rough estimate that 50% of designs with ten mutations would not contain a single deleterious mutation (**Fig 3B**). At this high TNR, the TPR is only 40%, reflecting the challenging tradeoff in the design of multipoint variants. We anticipate that in certain applications, such as in the design of improved antibodies for therapeutic application, a smaller number of mutations may be preferred. In such cases, a lower TNR and therefore a higher TPR may be implemented, and **Fig 3C** provides a guide for choosing different (PSSM, ΔΔ*G*) thresholds. Instructions for computing a mutation-tolerance map based on any structure of an antibody Fv are available as Supplemental Data, and the AbLIFT web server enables user control of these parameters.

### Automated affinity and stability design in the vL-vH interface

We next sought to develop a general and fully automated design protocol for improving molecular interactions across the vL-vH interface. AbLIFT starts by computing a mutational-tolerance map at the vL-vH interface using the approach described above; it then exhaustively enumerates the multipoint combinations of tolerated mutations; ranks them by energy, and selects low-energy variants for experimental testing. This algorithm resembles our recently described FuncLib method for designing functionally diverse enzyme repertoires [41], with the key differences that AbLIFT is applied to the core of obligatory binding surfaces rather than to solvent-exposed surfaces and most importantly, AbLIFT does not require an initial design round of protein stabilization.

To validate AbLIFT, we chose two antibodies as subjects for design: the synthetic antibody G6, which targets human Vascular-Endothelial Growth Factor (VEGF) [45], and an engineered variant of the 492.1 antibody, designated h492.1, which targets human Quiescin Sulfhydryl Oxidase 1 (QSOX1). QSOX1 is a multi-domain disulfide-catalyst that is overproduced in tumors [46] and is a potential drug target [47,48]. These antibodies are unrelated to D44.1 or to one another and are the products of protein engineering. G6 is widely used in animal studies and resulted from a phage-displayed synthetic Fab library of the light chain with a heavy chain sequence of an anti-mVEGF antibody (*K*_*D*_ approximately 1 nM) [49]. The h492.1 antibody was obtained by fusing the variable domains from the high-affinity (*K*_*D*_ approximately 1 nM) QSOX1-inhibiting murine antibody 492.1 onto a human IgG scaffold. Following this fusion, h492.1 could not be expressed to detectable levels in a recombinant cultured human cell system, frustrating its further development. Thus, with these two targets, we sought to test the ability of AbLIFT to optimize high-affinity antibodies that resulted from conventional antibody-engineering procedures, whether well-behaved ones (G6) or ones that showed low (or no) detectable expression levels (h492.1).

The computed mutational-tolerance map of G6 (starting from its bound structure, PDB entry 2FJG) at 30 vL-vH interface positions defined 26 affinity-enhancing mutations at 11 positions. To achieve significant improvement of vL-vH interface packing, we sought to design multipoint mutants with 4–10 mutations relative to G6, resulting in a tolerated sequence space of 203,835 unique multipoint mutants. All multipoint mutants were modeled in Rosetta, including by a backbone and side-chain minimization step, which is essential for enabling cavity-filling small-to-large mutations [50,51], and the models were then ranked by energy. 53% of the mutants (>100,000) exhibited energies as favorable as or better than the G6-bound antibody. Therefore, although the exhaustive enumeration of this large number of mutants is computationally demanding (approximately 6,000-CPU hours), the very large number of potentially improved designs makes a compelling case for exhaustive enumeration and ranking of variants within the tolerated sequence space. Furthermore, the computed mutational-tolerance map focuses exhaustive enumeration on a subset of stable multipoint mutants within the vast hypothetical sequence space of mutants at the vL-vH interface (20^30^ = 10^39^ unique sequences), >99% of which are predicted to have reduced stability compared to the parental antibody (**S3 Fig**).

**Fig 4:**
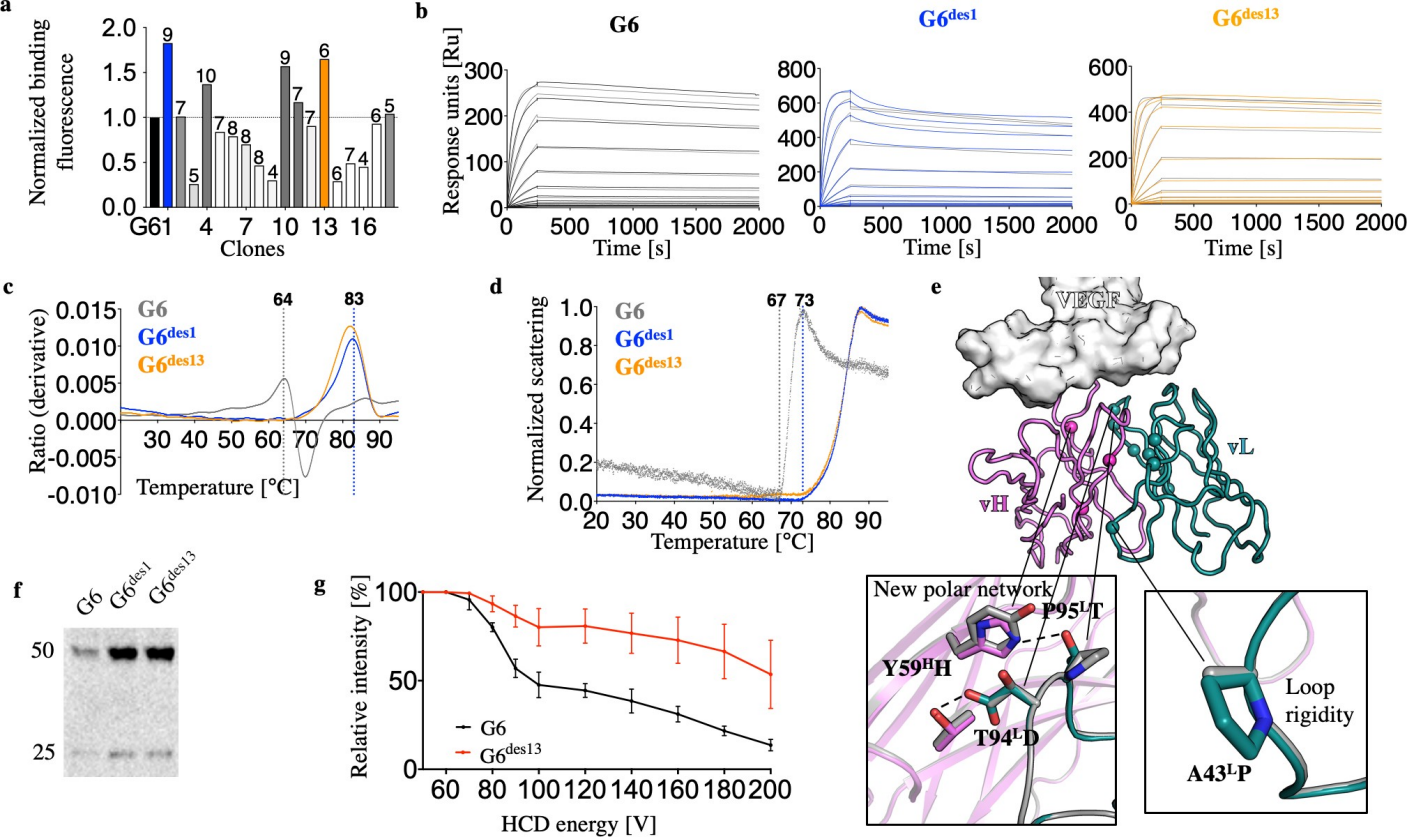
Fully automated antibody stability and affinity optimization using AbLIFT. (**a**) G6 and 18 low-energy designs, each encoding 4–10 mutations relative to G6 (number of mutations is indicated above the bars) were tested for binding using yeast display at 8 nM VEGF concentration, resulting in seven designs that showed comparable or higher binding signal compared to G6. G6^des1^ and G6^des13^ were chosen for further characterization (colored in blue and orange, respectively). (**b**) SPR kinetic analysis of VEGF binding with twofold dilutions from a maximal concentration of 100 nM by G6, G6^des1^, and G6^des13^ Fabs demonstrated faster binding on-rate in the designs (*k*_*a*_ = 2.3 * 10^5^ M^-1^s^-1^, 3.27 * 10^5^ M^-1^s^-1^ and 5.3 * 10^5^ M^-1^s^-1^, respectively). G6^des13^ also improved binding off-rate (*k*_*d*_ = 3.2 * 10^−5^ s^-1^ compared to 6 * 10^−5^ s^-1^ in G6), resulting in an improved dissociation constant (*K*_*D*_ = 60 pM compared to 270 pM in G6). (**c & d**) Thermal denaturation and temperature of aggregation onset experiments, respectively, using microscale thermophoresis indicated substantially higher apparent stability in the designs. (**e**) Computational mutation-tolerance mapping indicated 11 positions at the vL-vH interface of the anti-VEGF antibody G6 (spheres) with potentially tolerated mutations. Thumbnails indicate selected mutations in a model structure of G6^des13^ relative to G6 (gray). (**f**) Expression levels in HEK293 cells of G6 and the designs formatted as IgG were measured using Western blot analysis showing approximately an order of magnitude improvement in IgG expression levels for the designs. **(g)** Native mass-spectrometry analysis exhibited higher tolerance to applied collision energy in G6^des13^ compared to G6, both formatted as IgG. The error bars represent standard deviations inferred from three repeats.

We clustered the designs, eliminating ones that had fewer than four mutations relative to one another and selected the 18 lowest-energy ones for experimental testing. The designs were formatted as scFvs, and their binding signals relative to the G6 antibody were first qualitatively measured at 8 nM VEGF concentration using yeast display [3] (**Fig 4A**). Encouragingly, seven designs (approximately 40%) showed comparable or higher binding signal at this concentration. The best two designs, G6^des1^ and G6^des13^, were expressed as Fabs. When subjected to Ni-NTA purification, G6 exhibited multiple bands, indicative of sample heterogeneity, whereas, remarkably, both designs eluted mostly in the size expected for a Fab (**S4A and S4B Fig**) [52].

Next, the designs’ affinities for VEGF were determined using SPR (**Fig 4B**). Both designs improved binding on-rate, and G6^des13^ also improved the off-rate, resulting in fivefold improvement in *K*_*D*_ relative to G6. Both designs also exhibited substantial improvements in thermal stability and the temperature of aggregation onset (19° C and 10° C, respectively) (**Fig 4C and 4D**). We examined the model structure of G6^des13^, which comprised six mutations at the vL-vH interface relative to G6, finding that the mutations were likely to improve the interface through backbone rigidification and the introduction of a new buried polar hydrogen-bond network (**Fig 4E**). Such cooperative interaction networks do not typically arise in conventional antibody affinity-maturation processes (either in nature or the laboratory), which select mutations in a stepwise manner and are therefore biased towards additive rather than cooperative multipoint mutations. Introducing accurate new polar interaction networks is also a fundamental challenge for computational design [53,54] and the use of evolutionary constraints during design has recently been shown to overcome this challenge [42].

We next tested the stability and expressibility of the VEGF designs formatted as full-length IgGs. We expressed G6, G6^des1^, and G6^des13^ in HEK293 cells and found that the designs exhibited nearly an order of magnitude higher expression level than G6 (**Fig 4F**). We next measured the relative stabilities of G6 and G6^des13^ using native mass spectrometry [55] under reducing conditions by titrating the collision energy (**Fig 4G**). We found that G6 IgG disassembly started at lower collision energy compared to G6^des13^, indicative of the design’s higher stability (**S5 Fig**). We, therefore, concluded that AbLIFT could substantially improve expressibility, stability, and affinity, regardless of whether the antibody was formatted as Fab or IgG.

**Fig 5:**
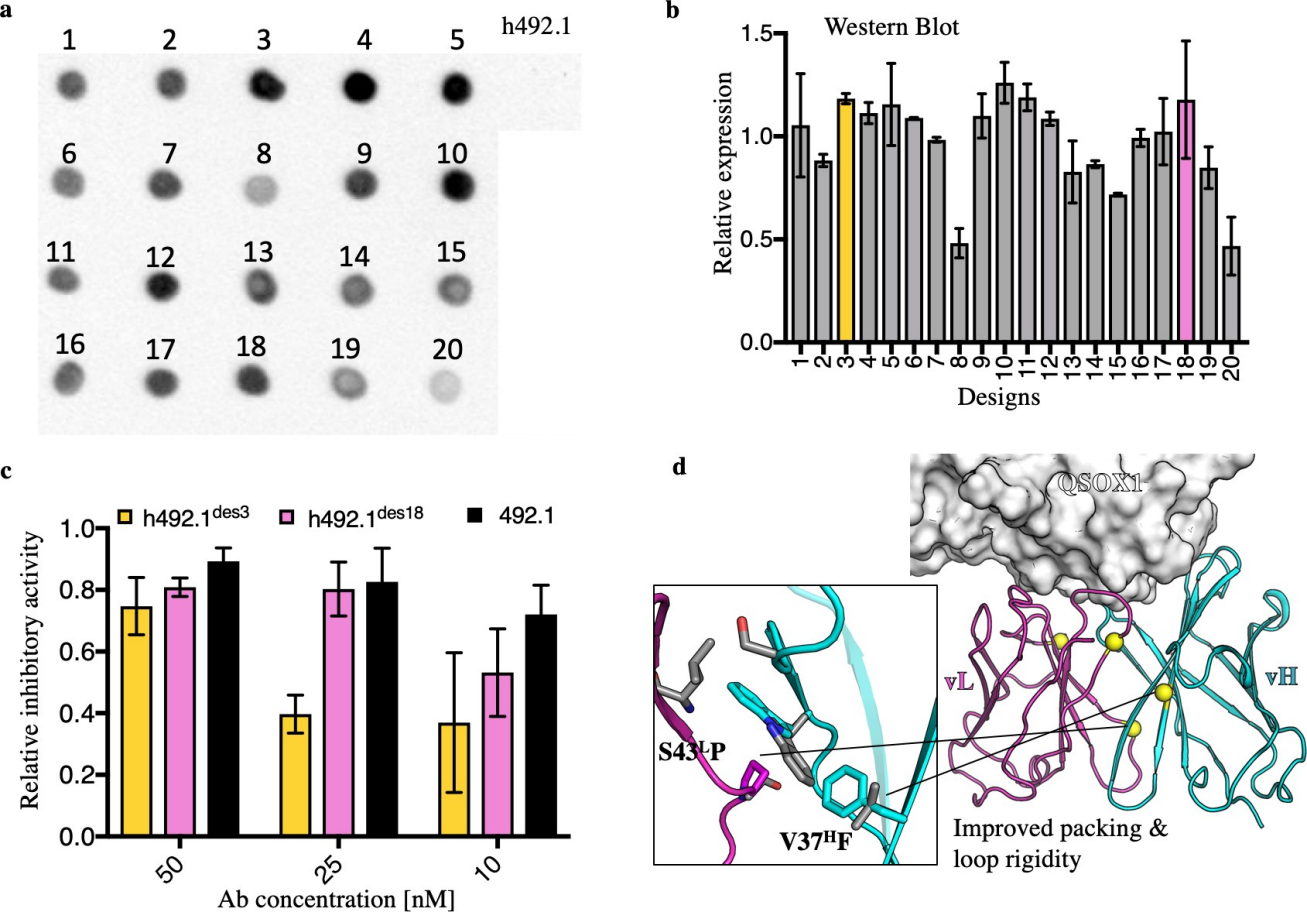
Substantial increase in antibody expression yields following AbLIFT design. (**a**) Dot blot analysis showed no detectable expression for h492.1 in HEK293 cells, whereas all 20 designs showed detectable levels of expression. (**b**) Relative expression levels of the 20 designs using Western blot analysis. h492.1^des3^ and h492.1^des18^ showed high expression and were selected for further analysis. (**c**) QSOX1 inhibitory activity assay using the parental 492.1 antibody and two designs. The inhibitory activity was measured for each antibody in a sulfhydryl oxidase assay using a physiological concentration of QSOX1 (25 nM). h492.1^des18^ showed comparable inhibitory activity relative to the parental antibody, with only a slight decrease when provided at sub-stoichiometric amounts (10 nM). (**d**) The structural context of mutations in h492.1^des18^ (color) relative to the experimental structure of 492.1 (gray). Spheres indicate the locations of the mutations, and the thumbnail shows two of the four designed mutations, which improve interchain packing and rigidify the backbone at the vL-vH interface according to the model structure.

We applied the same computational strategy to h492.1, in which the Fv was derived from a murine antibody and the constant regions were derived from human IgG1. Since h492.1 failed to show detectable expression in HEK293 cell cultures, we started the computational design from the structure of the murine 492.1 parental antibody in complex with QSOX1 (PDB entry: 4IJ3) [47]. We selected the 20 lowest-energy, sequence-clustered AbLIFT designs, fused them to human IgG1 constant domains and subjected them to HEK293-expression screening from crude cell lysate supernatant. Dot-blot analysis showed detectable expression levels for all 20 designs, in clear contrast with the lack of detectable expression for h492.1 (**Fig 5A**). We further quantitated expression levels using Western blot, revealing substantial variation in the expression levels among the designs (**Fig 5B**). In parallel, we examined the levels of QSOX1 inhibition by the 20 designs, finding that 50% showed high levels of QSOX1 inhibition (**S6 Fig**). Based on activities and expression levels, we selected h492.1^des3^ and h492.1^des18^ for further analysis. These designs were purified and added to QSOX1 activity assays to test for inhibition. h492.1^des18^ showed comparable inhibitory levels to the murine parent antibody when provided at equimolar amounts to a typical physiological concentration of QSOX1 (25 nM) as found in human serum (**Fig 5C**) [56]. This analysis demonstrated that h492.1^des18^ almost completely recovered the activity of the parental antibody while gaining high expression levels (approximately 75 mg/L supernatant). Structural analysis indicated that this design improved packing at the vL-vH interface (**Fig 5D**), demonstrating that in some cases optimizing this region could have a dramatic effect on the expression levels of engineered antibodies.

Finally, we asked whether there were any sequence features in common among the designs (**S3 and S4 Tables**). Strikingly, position 43^L^ (Chothia numbering) was mutated to Pro in D44.1^des^ and in >60% of the G6 and h492.1 designs. Position 43^L^ is located in a tight turn that connects two neighboring β strands, away from the CDRs, but Pro is not the consensus identity at this position (Ala and Ser are preferred). Furthermore, mutations at this position may have an important effect on the rigid-body angle formed by the variable light and heavy domains [44,57], and it is, therefore, unlikely that this mutation would universally improve antibody stability and affinity. Other than this mutation to Pro, we did not observe common sequence features among the designs. Overlapping but non-identical sets of positions were varied in each of the three case studies presented here, and the mutations at aligned positions were dissimilar. We, therefore, concluded that the designs improved interactions across the vL-vH interface through a variety of mechanisms that depended on the specific molecular structure of the parental antibodies.

## Discussion

Our study demonstrates that improved interactions across the vL-vH interface may result in substantial optimization of a range of essential parameters for antibody development, including expressibility, stability, and affinity. The automated AbLIFT strategy enables the design of cooperative networks of multipoint mutations in the antibody core that are likely to be inaccessible to experimental affinity maturation processes since these latter methods select mutations in a stepwise manner. Since AbLIFT impacts the antibody core and does not alter the structure of the antigen-binding site, the designed mutations cooperate with surface mutations identified through conventional antibody-engineering processes to further increase affinity and stability. AbLIFT may be particularly beneficial in antibodies, such as G6 and h492.1, which were the product of antibody-engineering approaches that might compromise antibody structural integrity, resulting in reduced affinity or stability. Moreover, antibody structure-prediction methods now often produce atomically accurate models at the vL-vH interface (though still not at the CDR H3) [58–60], suggesting that by restricting design to the framework regions, AbLIFT may in some cases enable antibody optimisation even in the absence of an experimental structure. We note, however, that AbLIFT considers only phylogenetic information and molecular energetics and disregards immunogenicity, which may be an important consideration in antibodies developed for clinical use. To address this concern, the AbLIFT web server enables complete control over the design sequence space and can be used to eliminate mutations with immunogenic potential.

The surprisingly broad ability of vL-vH design to optimize antibody properties is consistent with the Colman interface-adaptor hypothesis, according to which the formation of the Fv from two chains renders it flexible [34]. According to this hypothesis, Fv flexibility is likely to be an adaptive property selected by evolution to broaden molecular recognition by each individual antibody to a range of antigens through induced fit or conformational selection [61], thereby solving the conundrum of how a large but finite antibody repertoire could recognize a potentially infinite range of antigens [62]. Flexibility, however, might come at a cost, since an Fv that exhibits flexible vL-vH pairing may occupy multiple molecular states that compete with the binding-competent state, thus lowering antigen-binding affinity. Flexibility may moreover result in misfolding or transient dissociation of the two variable chains, resulting in terminal aggregation or degradation by the cellular proteostasis machinery, thereby lowering expression yields. In extreme cases, poorly defined packing at the vL-vH interface can lead to substantial rearrangements of the antibody variable domain during binding [63], and such rearrangements could lower antigen-binding affinity and specificity. Therefore, while the interface-adaptor hypothesis neatly explains why flexibility at the vL-vH interface is advantageous in early steps of antibody selection, broad specificity and marginal vL-vH interface stability become liabilities in later stages of antibody development into research or therapeutic tools. We anticipate that AbLIFT will have a wide scope to automatically and reliably improve stability, solubility, expressibility, affinity, and structural integrity in numerous antibodies in which these important properties are compromised.

## Methods

### D44.1 genetic library construction

Forward and reverse primers with the degenerate codon NNS were generated for all 135 positions on the Fv of D44.1, essentially as described [64]. Primers were ordered from Sigma (Sigma-Aldrich, Rehovot, Israel) and were used to introduce all possible amino acids per position by QuickChange mutagenesis [65]. Next, the PCR product of each position was transformed into yeast (EBY100 cells) and plated on SD-Trp as described [66]. Briefly, plates with more than 400 colonies were scraped with 1 ml SDCAA, 50 μl was added to 5 ml SDCAA tube and cells were then grown at 30°C overnight. The point mutants were split into six libraries, corresponding to positions that were at most 130 bp apart from one another to enable deep mutational scanning using 150 bp reads.

### Yeast surface display selection for libraries

Yeast-display experiments were conducted essentially as described [3]. Briefly, yeast cells were grown in selective medium SDCAA overnight at 30°C. The cells were then resuspended in 10 ml induction medium and incubated at 20°C for 20 h. 10^7^ cells were then used for yeast-cell surface display experiments: cells were subjected to primary antibody (mouse monoclonal IgG1 anti-c-Myc (9E10) sc-40, Santa Cruz Biotechnology) for expression monitoring and biotinylated ligand at 90 nM lysozyme (GeneTex) in PBS-F for 30 min at room temperature. The cells then underwent a second staining with fluorescently labeled secondary antibody (AlexaFluor488—goat-anti-mouse IgG1 (Life Technologies) for scFv labeling, Streptavidin-APC (SouthernBiotech) for ligand labeling) for 10 min at 4°C. Next, the cell fluorescence was measured and cells were collected under sorting conditions for expression and top 15% binders. The selection gates were calibrated using the wild-type scFv D44.1 and these gates were subsequently applied to the library constructs. Following fluorescence-activated cell sorting (FACS), cells were grown in SDCAA for 1–2 days and plasmids were extracted using Zymoprep Yeast Plasmid Miniprep II kit (Zymo Research).

### Yeast surface display of anti-VEGF scFvs

18 designs of improved binding affinity antibodies against VEGF and the wild-type G6 Fvs were ordered from Twist Biosciences as scFvs. These, as described above, were amplified by PCR and cloned into pCTCon2 using homologous recombination in yeast [66]. The plasmids were extracted by Zymoprep kit II, transformed into bacteria for sequence validation and verified clones were transformed to yeast for display [3]. The wild-type and designed antibodies were tested for binding by flow cytometry with 8 nM biotinylated VEGF (Recombinant Human VEGF 165, Biotinylated Protein R&D systems).

### DNA preparation for deep sequencing

To connect the DNA adaptors for deep sequencing, the plasmids extracted from the libraries were amplified using Phusion High-Fidelity DNA Polymerase (ThermoFisher) in a two-step PCR protocol.

PCR 1:

(barcode: CTCTTTCCCTACACGACGCTCTTCCGATCT)

>forward (seg1):**<barcode>**AGGGTCGGCTAGCCATATG

>forward (seg2):**<barcode>**GGATCGAATGGGTTAAACAACGT

>forward (seg3):**<barcode>**ACACCTCCTCTAACACCGC

>forward (seg4):**<barcode>**CTGGTGGCGGTGGCTC

>forward (seg5):**<barcode>**GCCGTGCGTCTCAGTCTATT

>forward (seg6): **<barcode>**CCATCTCGTTTCTCCGGC

>reverse: **CTGGAGTTCAGACGTGTGCTCTTCCGATCT**GGATCGAATGGGTTAAACAACGT

The PCR product for each population (expressed and top 15% of binders for each of the six libraries) was cleaned using Agencourt AMPure XP (Beckman Coulter, Inc.) and 1 μl from a 1:10 dilution was taken to the next PCR step for index labeling using KAPA Hifi DNA-polymerase (Kapa Biosystems, London, England):

>forward: AATGATACGGCGACCACCGAGATCTACACTCTTTCCCTACACGACGC

>reverse: CAAGCAGAAGACGGCATACGAGAT<index>GTGACTGGAGTTCAGACGTGTGC

Top 15%—index: CAATAGTC

Expressed—index: TTGAGCCT

All the primers were ordered as PAGE-purified oligos. The concentration of the PCR product was measured using Qu-bit assay (Life Technologies, Grand Island, New York).

### Deep-sequencing runs

DNA samples were run on an Illumina MiSeq using 150-bp paired-end kits. The FASTQ sequence files were obtained for each run, and customized scripts were used to generate the selection heat maps from the data as previously described [64]. Briefly, the script starts by translating the DNA sequence to amino acid sequence; eliminates sequences that harbor more than one amino acid mutation relative to wild type and also sequences that failed the QC test; counts each variant in each population; and eliminates variants with fewer than 100 counts in the reference population (to reduce statistical uncertainty).

### Sequencing analysis

To derive the mutational landscapes we compute the frequency *P^i,j^* of each mutant relative to wild-type in the selected and reference pools, where *i* is the position and *j* is the substitution, relative to wild-type:

$$P^{i,j}=\frac{{count}^{i,j}}{{count}_{wild-type}}$$ (Eq 1)

where count is the number of reads for each mutant. The selection coefficients are then computed as the ratio:

$$S^{i,j}=\frac{{\left(P^{i,j}\right)}_{selected}}{{\left(P^{i,j}\right)}_{reference}}$$ (Eq 2)

where *selected* refers to the top 15% binding population and reference refers to the reference population (Expression). The resulting *S^i,j^* values are then transformed to *−ln* enrichment values:

$$-ln(S^{i,j})$$ (Eq 3)

### Computational methods

All Rosetta design simulations used git version fb77c732b4f08b6c30572a2ef7760ad3bb4535ca of the Rosetta biomolecular modeling software, which is freely available to academics at http://www.rosettacommons.org. Position-Specific Scoring Matrices (PSSM) for designed antibodies against VEGF (PDB: 2FJG) and against QSOX1 (PDB: 4IJ3) were collected as described in ref. [38] and are distributed with the Rosetta release. RosettaScripts [67] and command lines are available in Supplemental Data. As in the *AbDesign* method [38], separate PSSMs were generated for CDRs 3 and for CDRs 1, 2 and the framework by aligning structurally similar antibodies in the PDB and selecting only sequences that did not exhibit gaps relative to the query sequence; furthermore, a strict cutoff of ≤ 0.5 Å backbone-carbonyl rmsd was used to eliminate structurally divergent sequences. Thus, the PSSMs were only based on structural considerations and not on sequence homology or source organism.

We refined each bound PDB structure by four iterations of side-chain packing and side-chain and backbone minimization, saving the minimum-energy structure. Computational mutation scanning was applied to the refined structure using the FilterScan filter in Rosetta [24]. At every position, each allowed mutation (that is, every amino acid identity with PSSM score ≥-1) was modeled singly against the background of the refined structure. Protein side chains within 8 Å of the modeled mutation were repacked, and side-chain and constrained backbone minimization were used to accommodate the mutation. The energy difference between the refined structure and the optimized configuration of the single-point mutant was calculated using the talaris2014 energy function [68]. The energy threshold used to define the tolerated mutation space was +1 R.e.u. We next enumerated all possible combinations of mutations against VEGF (203,835 models) and against QSOX1 (491,235 models), modeled them in Rosetta and relaxed them by sidechain packing and sidechain, backbone and rigid-body minimization with harmonic backbone coordinate restraints. Designs were ranked based on their energy and the top 18 designs differing by 4–10 mutations relative to one another (VEGF) (**S2 Table**) and the top 20 designs differing by 3–14 mutations relative to one another (QSOX1) (**S3 Table**) were selected for experimental characterization.

### The AbLIFT web-server

The web-server implements several improvements relative to the method used to design the G6 and h492.1 variants [41]. In the AbLIFT web-server, the multiple-sequence alignment used to construct the PSSM is first filtered to eliminate all loops and secondary-structure elements that exhibit any gaps relative to the query sequence. Furthermore, the web-server implements more accurate atomistic scoring and enables greater user control: it uses the recent Rosetta energy function ref15 [69] with improved electrostatics and solvation potentials relative to the previous Rosetta energy function talaris and allows the user to manually modify the tolerated sequence space (for instance, based on prior experimental data or to eliminate potential immunogenic sequence signatures). Accordingly, ΔΔ*G* and PSSM cutoffs may be different from those used to in the designs described in the paper, and the web server provides user control over these parameters.

### Bacterial expression and purification (D44.1 and D44.1^des^)

The design and wild-type were transformed into RH2.2 plasmid for expression as Fabs, where the heavy chain was N-terminally His-tagged and the light chain was expressed as a separate protein. Both chains contain a secretion sequence for direction to the periplasmic space, where they fold and dimerize. Restriction-free cloning was done using Kapa HiFi Hotstart Readymix (Kapa Biosystems) according to the manufacturer’s protocol.

Cells were induced with 1 mM IPTG at OD600 = 0.6, transferred to 20°C, and harvested after 20 h. The cells were then resuspended in buffer A [20 mM phosphate buffer pH 6.2, 150 mM NaCl] and sonicated. The supernatant was harvested by centrifugation (20,000 × *g*, 1 h), filtered, and loaded on HiTrap TALON crude 1 ml column (GE Healthcare). Then it was washed with 15–20 bed volumes of buffer A, and then eluted with buffer B [20 mM phosphate buffer pH 6.2,150 mM NaCl, 150 mM imidazole]. Imidazole was removed from the eluate by dialysis against Buffer C [20 mM Hepes buffer pH 7,150 mM NaCl] (1:400). The sample was then concentrated (Amicon Ultra-15 Centrifugal Filter; Merck) and purified by gel filtration in buffer C over a HiLoad 16/600 Superdex 200 pg column.

### Secreted IgG (G6, G6^des13^) and Fab (D44.1^des^) production in suspension

Antibodies were expressed in suspension-HEK293F cells, grown in FreeStyle medium (Gibco), in a shaking incubator (115 rpm), at 37°C, in a controlled environment of 8% CO_2_. The variable regions of the different heavy and light chains were cloned separately, upstream of IgG1 human Ab scaffolds, into p3BNC plasmids. Transfections were done using linear 40 kDa polyethyleneimine (PEI) (Polysciences) at 3 mg of PEI per 1 mg of plasmid DNA per 1 L of culture, at a cell density of 1 million cells/ml. Growth media were collected after 5–7 days and separated from cells by centrifugation at 600 x g. Media were then supplemented with 0.02% (wt/vol) sodium azide and 0.1 mM PMSF and further clarified by centrifugation at 16,840 x g for 30 min.

### Fab production (D44.1, G6, G6^des1^, G6^des13^)

Adherent HEK293T cells were cotransfected with genes encoding the light and heavy chain Fabs (heavy chain fused to C-terminal His tag) in p3BNC plasmids using linear PEI as a transfection reagent (12.5 μg/12.5 μg/50 μg, respectively, per 15-cm plate). Seventy-two hours post-transfection, the medium containing the secreted protein was collected (~250 ml).

### Fab purification (D44.1, D44.1^des^, G6, G6^des1^, G6^des13^)

The filtered medium was concentrated to ~200 ml using a diafiltration device (QuixStand Benchtop System; GE Healthcare). The medium composition was exchanged to buffer A [50 mM Tris pH 8 and 150 mM NaCl] using the same device. This was loaded on a HisTrap HP 5 ml column (GE Healthcare). Washed with 15 bed volumes of 20 mM Tris pH 8, 150 mM NaCl and 10mM imidazole and was eluted with 20 mM Tris pH 8, 150 mM NaCl and 250 mM imidazole. Imidazole was removed from the eluate by dialysis against Buffer A (1:400). The sample was then concentrated (Amicon Ultra-15 Centrifugal Filter; Merck) and purified by gel filtration in buffer A over a HiLoad 16/600 Superdex 200 pg column.

### Apparent *T*_*m*_ and aggregation onset measurements

The apparent melting temperature of the antibodies was determined by Prometheus NT. Plex instrument (NanoTemper Technologies), a label-free method. Fabs obtained from secreted Fab production in adherent cells (D44.1, G6, G6^des1^, G6^des13^) and from production in suspension (D44.1^des^) were diluted to 0.2 mg/ml (in 20 mM Hepes pH 7 and 50mM NaCl for anti-lysozyme antibodies and in 20 mM Hepes pH 7.5, 150 mM NaCl for anti VEGF antibodies). The temperature was ramped from 25°C to 100°C at 0.05°C/s, and both *T*_*m*_ and aggregation-onset temperature were measured.

### Surface-plasmon resonance

Surface plasmon resonance experiments on the anti-lysozyme (D44.1 and D44.1^des^ expressed in bacteria) and anti-VEGF antibodies (G6, G6^des1^ and G6^des13^ expressed in adherent cells) were carried out on a Biacore T200 instrument (GE Healthcare) at 25°C with HBS-N EP+ [10 mM Hepes, 150 mM NaCl, 3 mM EDTA, 0.005% vol/vol surfactant P20 (pH 7.4)]. For binding analysis, 1,000–1,600 response units (RU) of Fab were captured on a CM5 sensor chip. Samples of different protein concentrations were injected over the surface at a flow rate of 30 μL/min for 240 s, and the chip was washed with buffer for 2,000 s. If necessary, surface regeneration was performed with 30 s injection of 50 mM NaOH (D44.1^des^) or 10 mM NaOH (VEGF antibodies) at a flow rate of 30 μL/min. One flow cell contained no ligand and was used as a reference. The acquired data were analyzed using the device’s software, and kinetic fits were performed.

### IgG Western blot analysis (G6, G6^des1^, G6^des13^)

HEK293T cells were seeded on a 24-well plate pre-coated with poly-L-lysine at 120,000 cells/well. The next day, cells were transfected with 1 μg DNA mixture consisting of 200 ng pLXN plasmid encoding Luciferase, 400 ng of a plasmid encoding the light chains and 400 ng of a plasmid encoding the heavy chains of the designated antibodies. Each transfection was carried in 100 μl DMEM in which 2 μg of linear 40,000 Da PEI (Polysciences) per μg of DNA were mixed. The transfection mixture was added to cells, for a total volume of 400 μl DMEM per well. 4 hours after transfection, cells were washed and fresh 1 ml DMEM with 1% penicillin and streptomycin, glutamine and non-essential amino acids was applied. 72 hours post-transfection supernatant was separated from cells and the cells were resuspended in 500 μl PBS. A sample of 100 μl from the suspended cells from each well was transferred to 96-well white plates (Nunc) with 100 μl of Bright-Glo reagent (Promega) to quantify the level of luciferase as a proxy for the transfection efficiency. Adjusted volumes of supernatants based on the luciferase levels were loaded on a gradient gel (Bio-Rad) and run according to manufacturer's instructions. Semi-dry blotting was performed to a nitrocellulose membrane followed by blocking in 5% milk powder in TBST (0.1% Tween 20, 20 mM Tris pH 8.0, 150 mM sodium chloride) buffer for 30 min at room temperature. Donkey anti-human IgG conjugated to HRP (Abcam) was used to detect the human IgG scaffold for 1 h at room temperature.

### Mass spectrometry sample preparation

Following IgG production in suspension (as described above), clarified media were aliquoted, snap frozen in liquid nitrogen and stored at -80°C. On the day of the measurements, samples were thawed and buffer exchanged into 1 M ammonium acetate, pH 7, using Micro Bio-Spin 6 Columns (Bio-Rad). To break all disulfide bonds, antibodies were then reduced for 4 h at 37°C in the presence of 20 mM TCEP, followed by two consecutive buffer exchanges into 1 M and 150 mM ammonium acetate, respectively.

### Native-mass spectrometry

Nanoelectrospray ionization (nano-ESI) MS experiments were performed on a modified Q-Exactive Plus Orbitrap EMR (Thermo Fisher Scientific, Bremen, Germany) [70]. All spectra are shown without smoothing. The instrument was calibrated externally, using cesium iodide. Typically, an aliquot of 2 μl protein solution was loaded into a gold-coated nano-ESI capillary prepared in-house, as previously described [71], and sprayed into the instrument. Conditions within the mass spectrometer were adjusted to preserve noncovalent interactions. The source was operated in positive mode, the capillary voltage was set to 1.7 kV, the capillary temperature was 180°C and argon was used as the collision gas in the higher-energy collision-induced dissociation (HCD) cell. MS spectra were recorded at a resolution of 10,000 and HCD voltage was set to 50 V, at trapping gas pressure setting of 3.9, which corresponds to HV pressure of 1.04 x 10^−4^ mbar and UHV pressure of 2.35 x 10^−10^ mbar. Bent flatapole DC bias and axial gradient were set to 2 V and 25 V, respectively.

### Gas-phase stability assay

Antibody stability was monitored by tandem MS (MS/MS), at different HCD voltages. The 23^+^ charge state of the G6 and G6^des13^ antibodies was isolated in the quadrupole, with an isolation window of 20 m/z, and the transmitted ions were subjected to collision-induced dissociation in the HCD cell, at a gradient of accelerating voltages ranging between 50–200 V. The relative abundance of the full IgG’s and the dissociated light chains, recorded at the different HCD voltages, was determined by measuring their peak heights. The total intensity of the light chains was calculated as the sum of intensities of their corresponding charge states. In each experimental condition, the total intensities of all the measured species were summed and referenced as 100% intensity. The relative intensity of each species was then calculated as a percentage of the total intensity. The stability assay was performed six times. Error bars represent standard deviation.

### Anti-QSOX1 antibody production

The coding sequences for variable domains of antibody 492.1 were fused to human antibody constant regions [72]. Mutations were introduced by site-directed mutagenesis into the resulting hybrid antibody expression plasmids according to published procedures [73]. Plasmids were transfected into suspension-adapted suspension-HEK 293F cells. The day before transfection, cells were split to 0.7 x 10^6^ cells/ml. For parallel expression of the parent hybrid antibody and the 20 variants, transfections were performed using 0.5 μg of each plasmid (heavy and light Ab chains) mixed with 3 μg PEI Max reagent (Polysciences Inc.) and incubated 20 min in 24-well tissue culture trays prior to addition of 1 ml cells per well. Plates were then agitated vigorously in a tissue culture incubator/shaker to prevent cell settling. After 4 days, cultures were transferred to microfuge tubes, and cells were pelleted by centrifugation at 500 x g for 10 min. Supernatants were transferred to fresh microfuge tubes, from which aliquots were taken for quantification of antibody expression and activity. For purification of selected Ab designs, transfections were done in 40 ml volumes, and plasmid and PEI Max amounts were scaled up accordingly. Cultures were grown for 6 days, and Ab was purified from the supernatant by protein G affinity chromatography (GE Healthcare).

### QSOX1 dot blot and Western blot assays

Relative antibody concentrations were determined from culture supernatants by dot and Western blotting. Blotting was conducted in triplicate for each of two biological replicates. For dot blots, 2 μl of each supernatant was spotted onto nitrocellulose membranes. Membranes were then covered with a blocking solution of PBS containing 0.1% Tween (PBS-T) and 5% bovine serum albumin (BSA) and gently agitated for 1 h at room temperature. For western blots, 10 μl of each supernatant was applied with non-reducing gel loading buffer to 10% SDS polyacrylamide gels. After electrophoresis, proteins were transferred to nitrocellulose, and the membranes were incubated in PBS-T with 5% BSA under gentle agitation. For both dot and Western blots, horseradish peroxidase-conjugated antibody recognizing human Fc was added to the blocking solution after the first hour, and incubation/shaking was continued for another 45 min. The membrane was then washed three times for 5 min each with PBS-T, and the blot was developed using SuperSignal West Pico (ThermoFisher) chemiluminescent substrate. Dot and band intensities were recorded on a ChemiDoc XRS+ system (Bio-Rad).

### QSOX1 inhibition assays

QSOX1 inhibition assays were conducted by using 5,5-dithio-bis-2-nitrobenzoic acid (DTNB) to quantify the remaining dithiothreitol (DTT) after incubation with purified recombinant QSOX1 and HEK293 culture supernatants or purified antibody. Culture supernatants (25 μl) were mixed in a clear, flat-bottom, 96-well plate with 12.5 μl of 40 nM QSOX1, and reactions were initiated by injection of 12.5 μl 600 μM DTT (final concentrations 10 nM QSOX1 and 150 μM DTT). Reactions were stopped after 30 min by adding 150 μl 500 μM DTNB, and absorbance at 412 nm was measured after 5 min in a Tecan microplate reader.

Purified antibody variants were quantified by absorbance at 280 nm after dilution into 6 M guanidine dissolved in PBS, using an extinction coefficient of 187,000 M^-1^cm^-1^. Purified antibodies (12.5 μl) at concentrations of 40 nM, 100 nM, and 200 nM were mixed in a 96-well plate with 12.5 μl 100 nM QSOX1, and reactions were initiated by injection of 25 μl 600 μM DTT (final concentrations 25 nM QSOX1, 300 μM DTT, and 10, 25, or 50 nM antibody). Reactions were stopped after 20 min by adding 150 μl 500 μM DTNB, and absorbance at 412 nm was measured after 5 min. Background-subtracted absorbance readings were normalized relative to the uninhibited and fully inhibited reactions (the latter mimicked by leaving QSOX1 out of the reaction), and results were plotted in Fig 5C as the relative inhibitory activity.

## Supporting information

S1 Fig **a. Mutational tolerance mapping of the anti-lysozyme antibody D44.1.** Mutations that were enriched, depleted, or had insufficient data in deep sequencing are marked in blue, red, and gray respectively. Wild type amino acids are indicated in one-letter codes for each position. Disulfide-bonded cysteines are marked in black triangles, and light-heavy chain interface positions in which point mutations exhibited over threefold enrichment relative to wild type, are marked in pink triangles. **b.** Qualitative binding titrations using yeast display for D44.1, D44.1^des^, and seven point mutants that comprise D44.1des using yeast surface display. Binding fluorescence intensities are relative to the highest concentration of 1 μM lysozyme.
(TIF)

S2 Fig **a.** The crystal structure of D44.1^des^ (yellow and green for heavy and light chains, respectively) shows high accuracy relative to the computational design (lavender). Electron density at 2 σ. **b.** Crystallographic analysis of D44.1^des^ shows high agreement with D44.1 (0.7 Å Cα root-mean-square deviation), including in the orientations of binding-surface residues (sticks; D44.1 in gray).
(TIF)

S3 Fig Computational mutation tolerance mapping enriches for low-energy designs. (blue) the distribution of Rosetta energies relative to G6 of a selection of >150,000 unique multipoint mutants at 11 positions encoded in the tolerated sequence space computed by PSSM (≥-1) and ΔΔ*G* (≤+1 R.e.u.) filters. (green) a random set of multipoint mutants at 30 vL-vH interface (all interface positions were allowed), where any of the 19 amino acid mutations was allowed at each mutated position. In both sets, the same number of multipoint mutants was analyzed, and the same distribution of the number of mutations relative to G6 was implemented. 37% of the multipoint mutants had energies that were more favorable than G6, whereas less than 0.03% of the random mutants had more favorable energies than G6. Thus computational mutation tolerance mapping enriches for improved mutants by over 1,100-fold relative to random multipoint mutations.
(TIF)

S4 Fig G6, G6^des1^, and G6^des13^ Fab expression and purification. (a) Following Ni-NTA purification, G6 exhibits the expected band at 50 kDa, and additional bands at approximately 100 kDa, indicative of sample heterogeneity. G6^des13^ and G6^des1^, by contrast, primarily elute at the 50 kDa size range with no detectable higher-mass bands. (b) Designs G6^des13^ and G6^des1^ after gel filtration run at their expected sizes. The status of reducing conditions (without DTT and boiling) is indicated at the bottom of the gels.
(TIF)

S5 Fig Secreted full-length IgG1 G6 and G6^des13^ antibodies were reduced and analyzed by native mass-spec directly from the growth medium. Upper panels show the full spectra. Charge state series of the two antibodies are labeled by dark blue and light blue circles, respectively. The +23 charge state of each antibody was isolated in the quadrupole and subjected to a gradual elevation of collision voltage in a stepwise manner, ranging from 50 to 200 V. Light chains, which gradually dissociated from the intact antibodies, are labeled the by red and orange circles.
(TIF)

S6 Fig All 20 h492.1 designs were expressed, and their activities from culture supernatants were measured as described in the methods. The highest values in the blot reflect the greatest amounts of substrate remaining at the end of a QSOX1 sulfhydryl oxidase activity assay, indicating the greatest inhibition of QSOX1 by the antibody. Due to differences in expression levels (Fig 5A and 5B), inhibitory activity in this experiment reflects a combination of expression yield and intrinsic activity. The designs with results plotted in color (yellow and pink) were expressed in larger volumes, purified, and compared quantitatively for inhibitory activity compared to the parental 492.1 antibody purified from a hybridoma (Fig 5C).
(TIF)

S1 Table Data collection and refinement statistics for D44.1des, PDB code 6GC2. (XLSX)

S2 Table The mutated positions and identities in G6 designs, colored according to their physicochemical properties and sorted by normalized fluorescence value (measured by yeast display experiments). (DOCX)

S3 Table The mutated positions and identities in anti-QSOX1 492.1 designs, colored according to their physicochemical properties. (DOCX)

S4 Table Log-enrichment of the deep mutational scanning data of anti-lysozyme antibody D44. Data retrieved from the deep mutational scanning analysis of enrichment over WT for single point substitutions.
(XLSX)

S1 Protocol RosettaScript for refinement of structures retrieved from the PDB. (TXT)

S2 Protocol RosettaScript for single-point mutational scanning. (TXT)

S3 Protocol RosettaScript for combinatorial sequence design. An example of a protocol for designing a specific combinatorial mutant.
(TXT)

S1 Text DNA sequences of tested constructs. (DOCX)

S2 Text Amino acid sequences of G6 and G6des13 IgGs. Protein sequences used in the mass spectrometry analyses.
(DOCX)
