# Correction: Optimizing antibody affinity and stability by the automated design of the variable light-heavy chain interfaces

**DOI:** 10.1371/journal.pcbi.1008382

**Published:** 2020-10-21

**Authors:** Shira Warszawski, Aliza Borenstein Katz, Rosalie Lipsh, Lev Khmelnitsky, Gili Ben Nissan, Gabriel Javitt, Orly Dym, Tamar Unger, Orli Knop, Shira Albeck, Ron Diskin, Deborah Fass, Michal Sharon, Sarel J. Fleishman

The funding statement for this article should read as follows:

“The research was supported by grants from the European Research Council (335439 to SJF, 636752 to MS, and 310649 and 825076 to DF), the Israel Science Foundation to MS (300/17) and through its Center of Excellence in Structural Cell Biology to SJF and DF (1775/12), a research grant from Sheri and David E. Stone and by a charitable donation from Sam Switzer and family. M.S. is an incumbent of the Aharon and Ephraim Katzir Memorial Professorial Chair. The funders had no role in study design, data collection and analysis, decision to publish, or preparation of the manuscript.”
