# Supplementary figures and images for: Optimizing antibody affinity and stability by the automated design of the variable light-heavy chain interfaces
Source: PLoS Comput Biol. 2019 Aug 23;15(8):e1007207. doi: 10.1371/journal.pcbi.1007207 (PMC6728052; doi:10.1371/journal.pcbi.1007207)

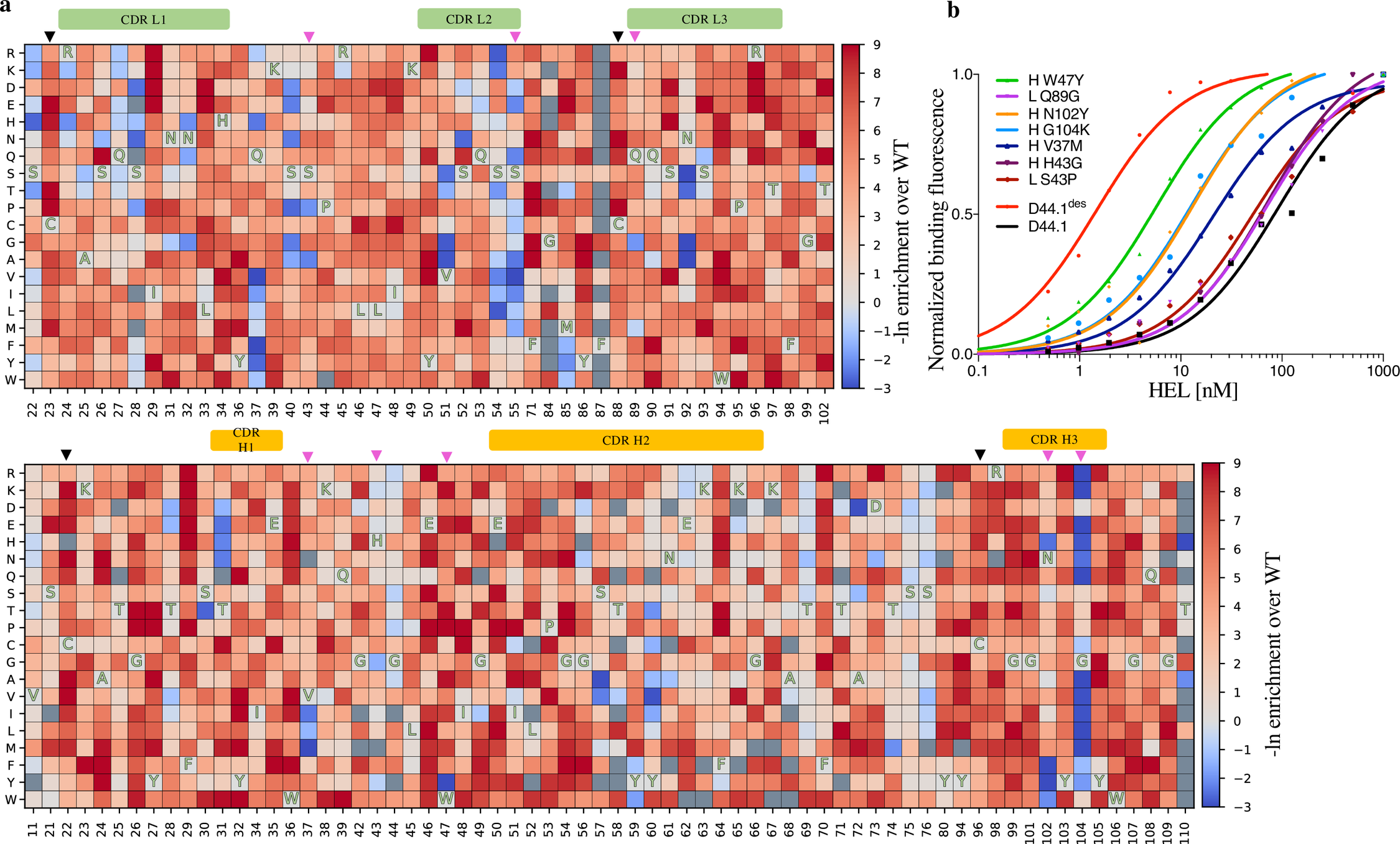

Supplement: S1 Fig — a. Mutational tolerance mapping of the anti-lysozyme antibody D44.1. Mutations that were enriched, depleted, or had insufficient data in deep sequencing are marked in blue, red, and gray respectively. Wild type amino acids are indicated in one-letter codes for each position. Disulfide-bonded cysteines are marked in black triangles, and light-heavy chain interface positions in which point mutations exhibited over threefold enrichment relative to wild type, are marked in pink triangles. b. Qualitative binding titrations using yeast display for D44.1, D44.1des, and seven point mutants that comprise D44.1des using yeast surface display. Binding fluorescence intensities are relative to the highest concentration of 1 μM lysozyme. (TIF) [file pcbi.1007207.s001.tif]

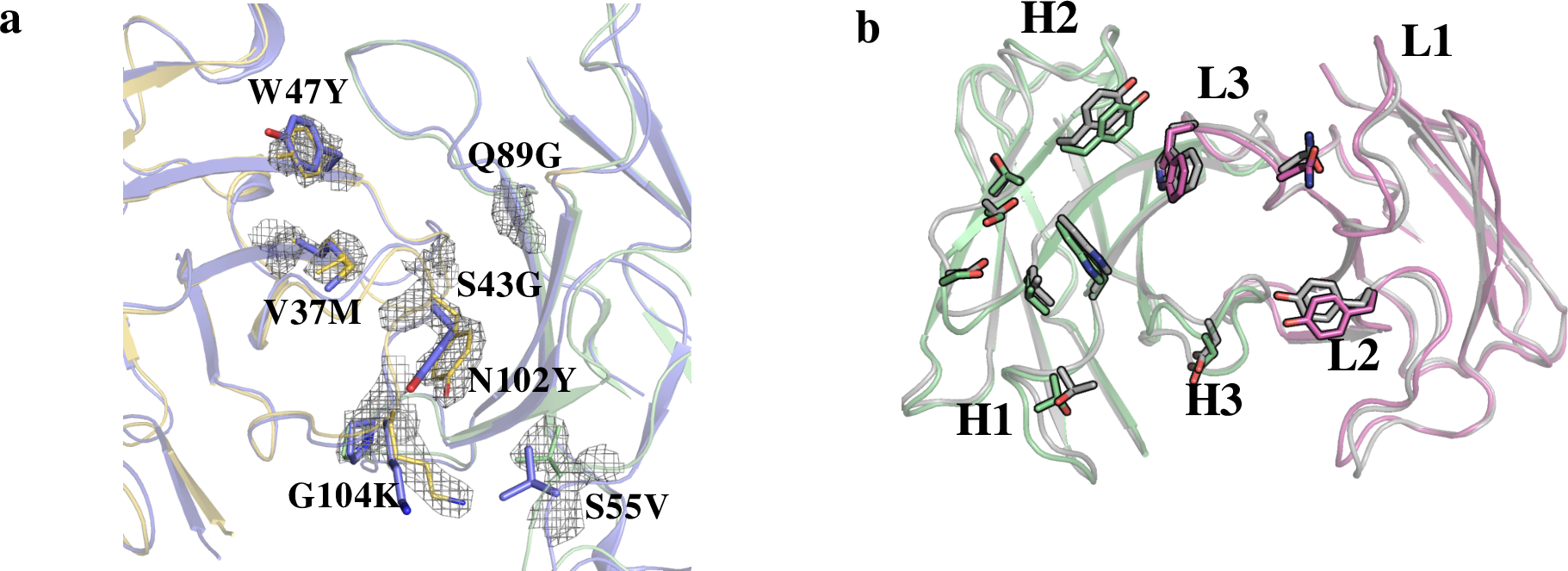

Supplement: S2 Fig — a. The crystal structure of D44.1des (yellow and green for heavy and light chains, respectively) shows high accuracy relative to the computational design (lavender). Electron density at 2 σ. b. Crystallographic analysis of D44.1des shows high agreement with D44.1 (0.7 Å Cα root-mean-square deviation), including in the orientations of binding-surface residues (sticks; D44.1 in gray). (TIF) [file pcbi.1007207.s002.tif]

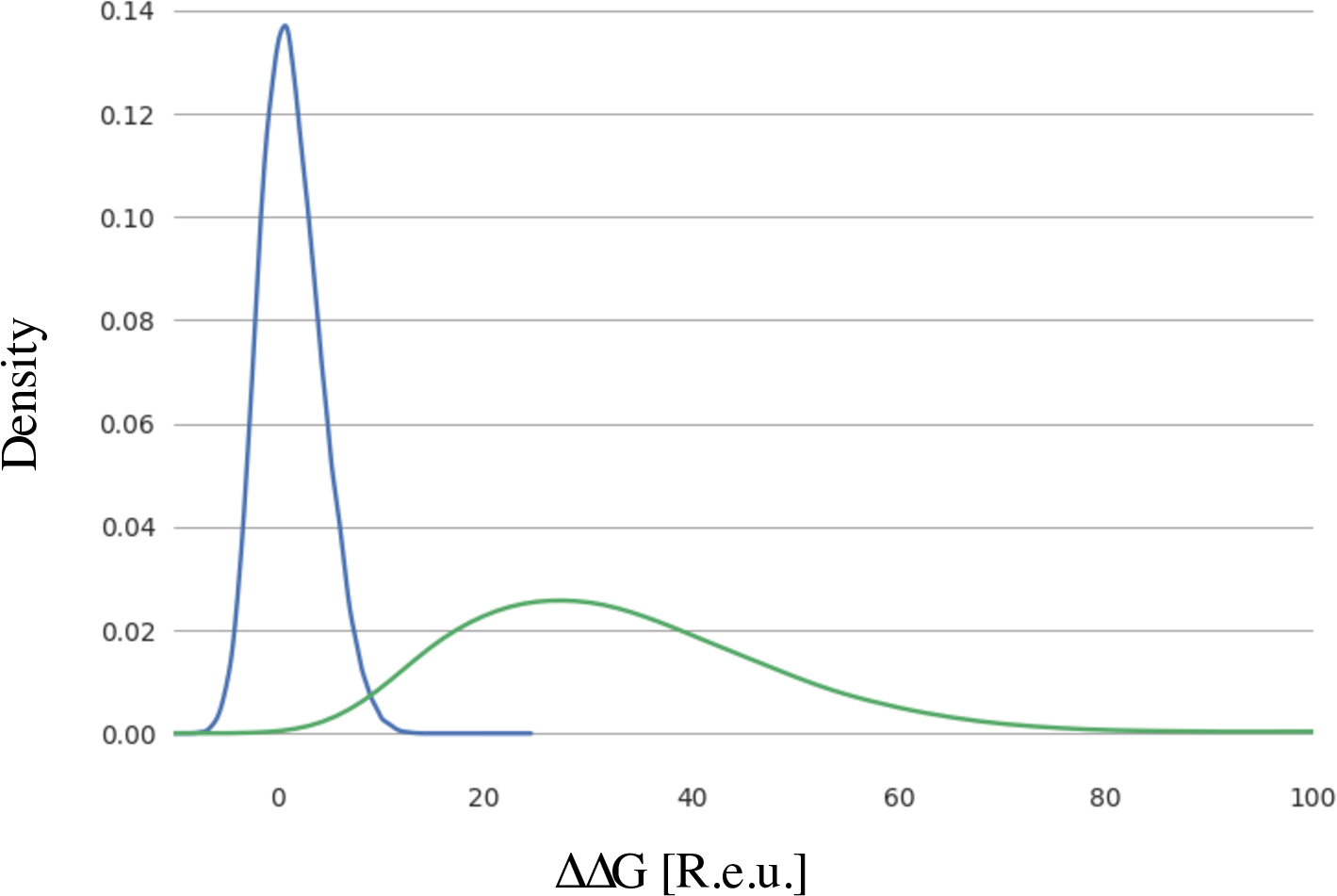

Supplement: S3 Fig — (blue) the distribution of Rosetta energies relative to G6 of a selection of >150,000 unique multipoint mutants at 11 positions encoded in the tolerated sequence space computed by PSSM (≥-1) and ΔΔG (≤+1 R.e.u.) filters. (green) a random set of multipoint mutants at 30 vL-vH interface (all interface positions were allowed), where any of the 19 amino acid mutations was allowed at each mutated position. In both sets, the same number of multipoint mutants was analyzed, and the same distribution of the number of mutations relative to G6 was implemented. 37% of the multipoint mutants had energies that were more favorable than G6, whereas less than 0.03% of the random mutants had more favorable energies than G6. Thus computational mutation tolerance mapping enriches for improved mutants by over 1,100-fold relative to random multipoint mutations. (TIF) [file pcbi.1007207.s003.tif]

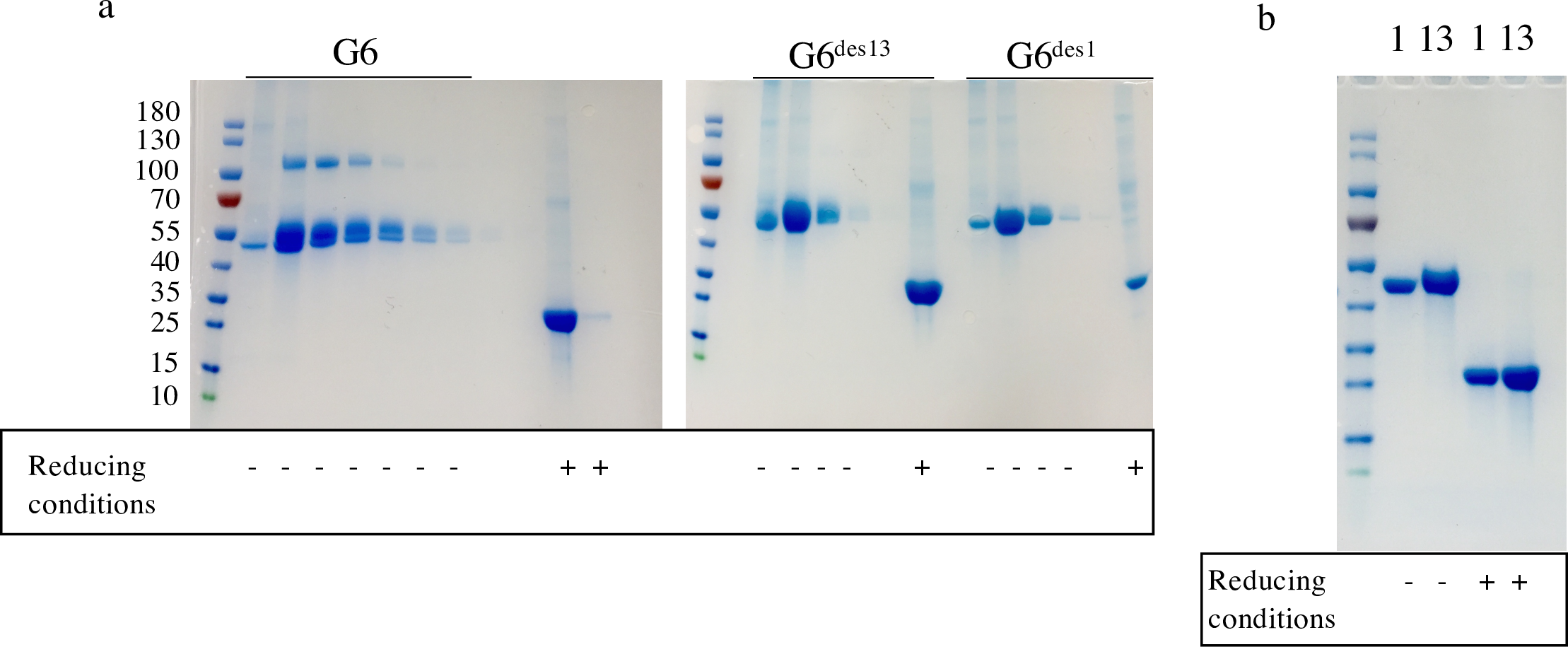

Supplement: S4 Fig — (a) Following Ni-NTA purification, G6 exhibits the expected band at 50 kDa, and additional bands at approximately 100 kDa, indicative of sample heterogeneity. G6des13 and G6des1, by contrast, primarily elute at the 50 kDa size range with no detectable higher-mass bands. (b) Designs G6des13 and G6des1 after gel filtration run at their expected sizes. The status of reducing conditions (without DTT and boiling) is indicated at the bottom of the gels. (TIF) [file pcbi.1007207.s004.tif]

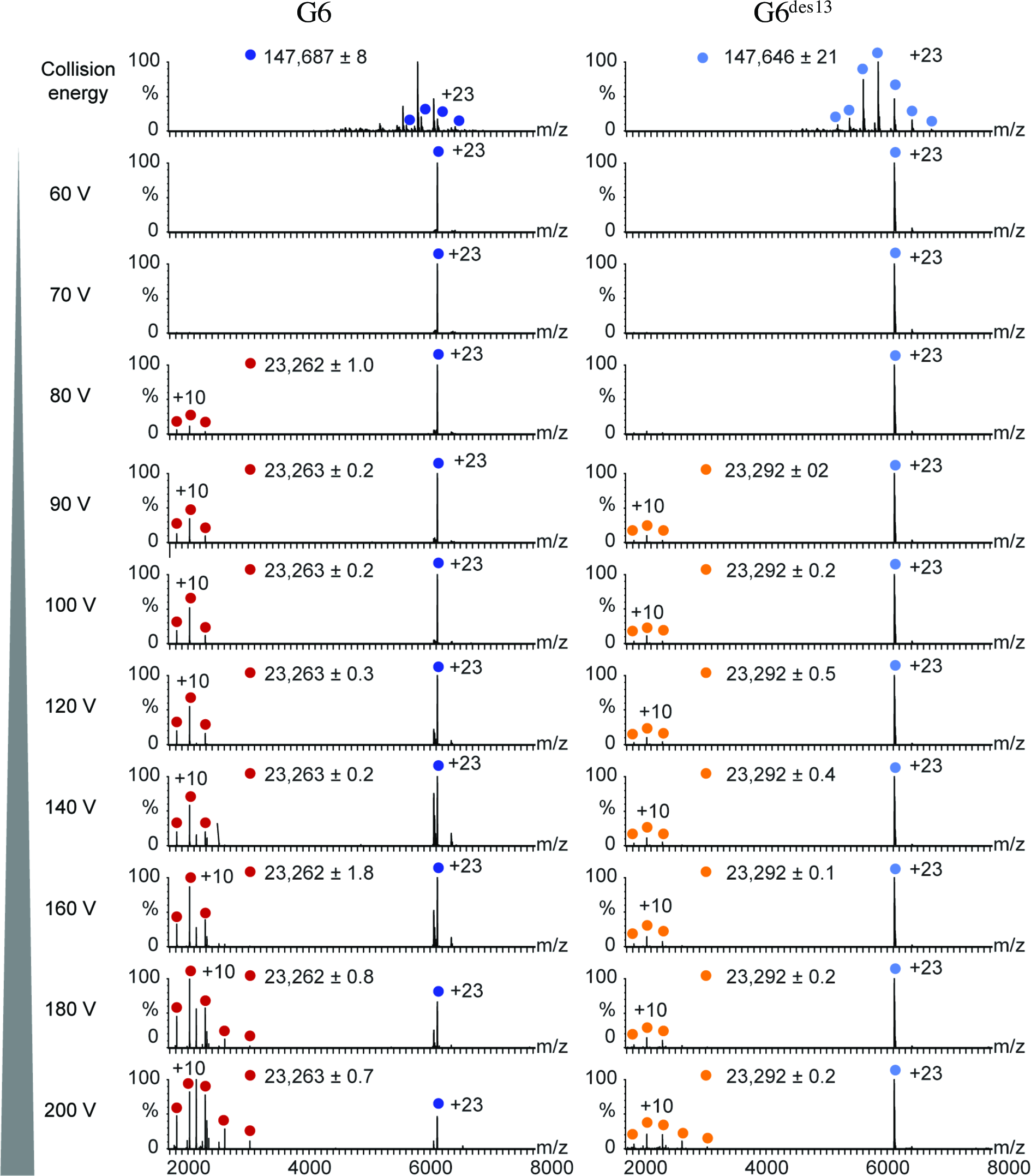

Supplement: S5 Fig — Upper panels show the full spectra. Charge state series of the two antibodies are labeled by dark blue and light blue circles, respectively. The +23 charge state of each antibody was isolated in the quadrupole and subjected to a gradual elevation of collision voltage in a stepwise manner, ranging from 50 to 200 V. Light chains, which gradually dissociated from the intact antibodies, are labeled the by red and orange circles. (TIF) [file pcbi.1007207.s005.tif]

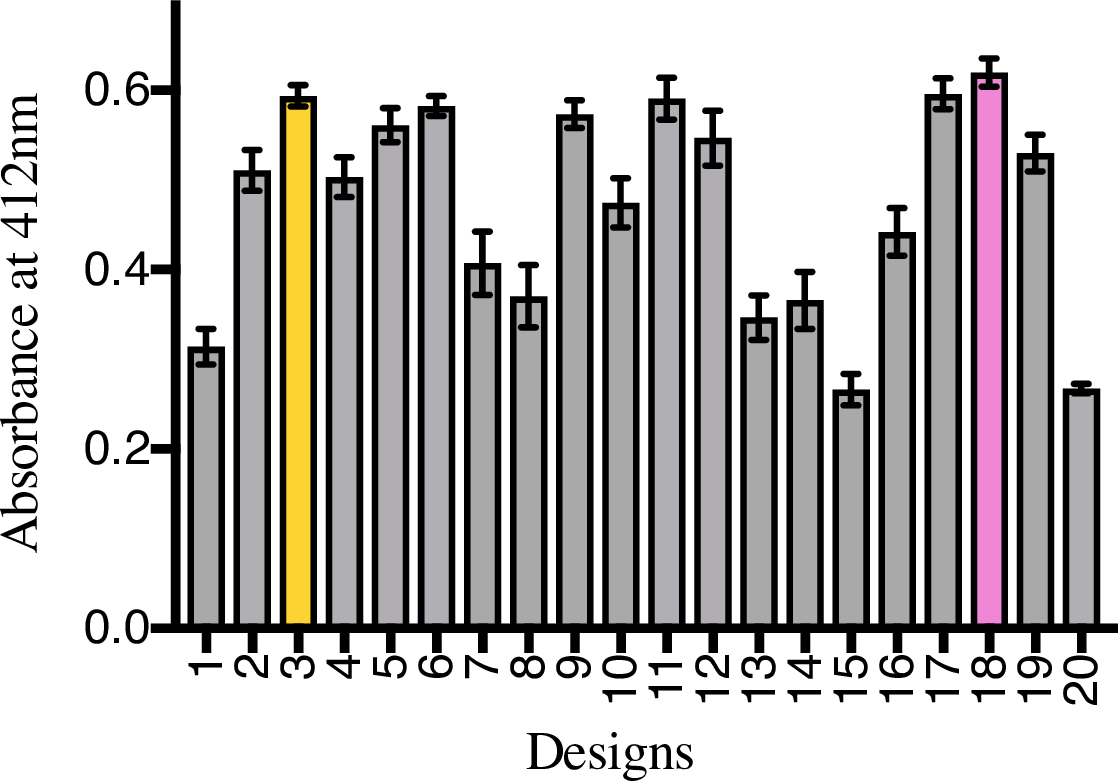

Supplement: S6 Fig — The highest values in the blot reflect the greatest amounts of substrate remaining at the end of a QSOX1 sulfhydryl oxidase activity assay, indicating the greatest inhibition of QSOX1 by the antibody. Due to differences in expression levels (Fig 5A and 5B), inhibitory activity in this experiment reflects a combination of expression yield and intrinsic activity. The designs with results plotted in color (yellow and pink) were expressed in larger volumes, purified, and compared quantitatively for inhibitory activity compared to the parental 492.1 antibody purified from a hybridoma (Fig 5C). (TIF) [file pcbi.1007207.s006.tif]
