# Supplementary material for: Optimizing antibody affinity and stability by the automated design of the variable light-heavy chain interfaces
Source: PLoS Comput Biol. 2019 Aug 23;15(8):e1007207. doi: 10.1371/journal.pcbi.1007207 (PMC6728052; doi:10.1371/journal.pcbi.1007207)
Supplement: S2 Table — (DOCX) [file pcbi.1007207.s008.docx]

**Table S2.** The mutated positions and identities in G6 designs, colored according to their physicochemical properties and sorted by normalized fluorescence value (measured by yeast display experiments).

| Position | L A43 | L Y49 | L Y55 | L Q89 | L T94 | L P95 | L T97 | L Q100 | H Y59 | H Y95 | H M107 | Normalized fluorescence [a.u] | Total score [R.e.u] |
| --- | --- | --- | --- | --- | --- | --- | --- | --- | --- | --- | --- | --- | --- |
| G6^des1^ | P | F | F | L | V | T | - | P | H | F | - | 1.83 | -616 |
| G6^des13^ | P | - | - | L | D | T | - | - | H | F | - | 1.65 | -616 |
| G6^des10^ | P | - | F | M | D | T | M | P | H | F | - | 1.57 | -615 |
| G6^des4^ | P | F | F | - | N | T | E | P | H | F | Y | 1.37 | -617 |
| G6^des11^ | S | - | F | L | - | T | M | - | H | F | - | 1.17 | -615 |
| G6^des18^ | P | - | - | - | I | T | - | - | H | - | H | 1.04 | -617 |
| G6^des2^ | - | F | - | - | N | T | E | P | H | - | H | 1.01 | -616 |
| **G6** | **A** | **Y** | **Y** | **Q** | **T** | **P** | **T** | **Q** | **Y** | **Y** | **M** | **1** | **-608** |
| G6^des17^ | P | - | F | - | M | T | - | - | - | F | H | 0.93 | -615 |
| G6^des12^ | P | - | - | L | I | T | E | - | - | F | H | 0.91 | -615 |
| G6^des5^ | P | F | - | L | N | T | - | P | - | F | H | 0.84 | -615 |
| G6^des6^ | P | F | - | - | D | T | E | - | H | F | H | 0.79 | -616 |
| G6^des7^ | P | F | F | - | D | T | - | P | H | H | - | 0.70 | -617 |
| G6^des15^ | P | - | F | - | N | - | - | P | F | F | Y | 0.49 | -617 |
| G6^des8^ | N | F | - | - | V | T | - | P | H | F | H | 0.47 | -615 |
| G6^des16^ | - | - | F | - | - | T | - | P | - | - | H | 0.45 | -615 |
| G6^des9^ | - | F | - | - | - | T | - | - | - | F | H | 0.30 | -616 |
| G6^des14^ | S | - | - | - | I | T | - | P | - | F | H | 0.29 | -616 |
| G6^des3^ | P | F | - | L | V | T | - | - | - | - | - | 0.26 | -616 |
