# Supplementary material for: Optimizing antibody affinity and stability by the automated design of the variable light-heavy chain interfaces
Source: PLoS Comput Biol. 2019 Aug 23;15(8):e1007207. doi: 10.1371/journal.pcbi.1007207 (PMC6728052; doi:10.1371/journal.pcbi.1007207)
Supplement: S3 Table — (DOCX) [file pcbi.1007207.s009.docx]

**Table S3.** The mutated positions and identities in anti-QSOX1 492.1 designs, colored according to their physicochemical properties.

|  | L S43 | L Y55 | L Q89 | L L96 | H V37 | H G44 | H L45 | H D58 | H F94 | H S104 | H F105 | H A106 | Total score [R.e.u] |
| --- | --- | --- | --- | --- | --- | --- | --- | --- | --- | --- | --- | --- | --- |
| **h492.1** | **S** | **Y** | **Q** | **L** | **V** | **G** | **L** | **D** | **F** | **S** | **F** | **A** | **-905** |
| h492.1^des1^ | - | H | - | F | - | A | - | - | Y | A | L | - | -916 |
| h492.1^des2^ | - | P | - | F | - | - | - | E | - | A | - | E | -916 |
| h492.1^des3^ | A | P | - | - | - | - | - | E | Y | A | L | E | -916 |
| h492.1^des4^ | P | H | - | - | - | - | - | E | Y | - | - | P | -916 |
| h492.1^des5^ | P | P | - | F | - | - | - | - | - | - | - | N | -916 |
| h492.1^des6^ | P | P | - | F | - | - | - | - | - | A | L | E | -917 |
| h492.1^des7^ | P | P | - | F | - | - | - | E | Y | - | L | E | -916 |
| h492.1^des8^ | P | P | - | F | - | A | - | - | - | - | Y | Q | -916 |
| h492.1^des9^ | P | P | - | F | - | A | - | - | Y | A | L | Q | -916 |
| h492.1^des10^ | P | P | - | F | - | A | - | E | - | - | L | - | -916 |
| h492.1^des11^ | P | P | H | - | - | - | - | - | - | A | - | Q | -917 |
| h492.1^des12^ | P | P | H | - | - | A | - | - | Y | - | - | Q | -916 |
| h492.1^des13^ | P | P | H | F | - | - | - | - | - | - | L | - | -916 |
| h492.1^des14^ | P | P | H | F | - | - | - | - | Y | A | Y | E | -916 |
| h492.1^des15^ | P | P | H | F | - | - | - | E | - | - | Y | Q | -916 |
| h492.1^des16^ | P | V | - | F | - | - | F | - | - | - | Y | Q | -916 |
| h492.1^des17^ | P | V | - | F | - | A | - | - | - | A | L | - | -917 |
| h492.1^des18^ | P | V | - | F | F | - | - | - | - | - | - | - | -916 |
| h492.1^des19^ | P | V | H | - | - | A | - | E | Y | A | L | - | -916 |
| h492.1^des20^ | P | V | H | F | - | A | - | - | - | - | Y | - | -916 |
