# Supplementary material for: Optimizing antibody affinity and stability by the automated design of the variable light-heavy chain interfaces
Source: PLoS Comput Biol. 2019 Aug 23;15(8):e1007207. doi: 10.1371/journal.pcbi.1007207 (PMC6728052; doi:10.1371/journal.pcbi.1007207)
Supplement: S1 Text — (DOCX) [file pcbi.1007207.s014.docx]

**DNA sequences of tested constructs**

>D44.1 (pCTcon2)

CAGGTTCAGCTGCAGGAATCTGGTGCGGAAGTTATGAAGCCGGGTGCGAGCGTGAAGATCTCCTGCAAAGCAACTGGTTACACCTTCTCCACCTATTGGATCGAATGGGTTAAACAACGTCCGGGTCACGGTCTGGAATGGATCGGTGAAATCCTGCCGGGCTCTGGTAGCACCTACTACAACGAAAAATTCAAAGGCAAAGCGACCTTCACCGCAGACACCTCCTCTAACACCGCGTATATGCAGCTCTCTTCTCTGACCTCTGAAGACAGCGCCGTTTACTACTGCGCGCGTGGCGACGGTAACTACGGTTACTGGGGCCAGGGTACCACCCTCACCGTTTCTTCCGCGTCCGGTGGCGGTGGTAGCGGTGGTGGTGGTTCTGGTGGCGGTGGCTCTGACATCGAACTCACTCAATCTCCAGCGACCCTCAGCGTTACCCCTGGTGACTCTGTGTCTCTGTCTTGCCGTGCGTCTCAGTCTATTTCTAACAATCTGCATTGGTATCAGCAAAAAAGCCATGAATCCCCTCGTCTGCTGATCAAATACGTTTCCCAATCTTCTTCTGGTATCCCATCTCGTTTCTCCGGCTCCGGCAGCGGCACTGACTTTACCCTGTCTATCAACTCCGTCGAGACGGAAGACTTCGGTATGTACTTCTGCCAGCAGAGCAACTCTTGGCCGCGTACCTTTGGCGGCGGTACCAAACTGGAAATCAAACGTCTCGAGGGCGGATCCGAACAAAAGCTTATTTCTGAAGAGGACTTGTAATAG

>D44.1^des^ (pCTcon2)

CAGGTTCAGCTGCAGGAAAGCGGCGCGGAAGTGATGAAACCGGGCGCGAGCGTGAAAATTAGCTGCAAAGCGACCGGCTATACCTTTAGCACCTATTGGATTGAATGGATGAAACAGCGCCCGGGCGGCGGCCTGGAATATATTGGCGAAATTCTGCCGGGCAGCGGCAGCACCTATTATAACGAAAAATTTAAAGGCAAAGCGACCTTTACCGCGGATACCAGCAGCAACACCGCGTATATGCAGCTGAGCAGCCTGACCAGCGAAGATAGCGCGGTGTATTATTGCGCGCGCGGCGATGGCTATTATAAATATTGGGGCCAGGGCACCACCCTGACCGTGTCTTCCGCGTCCGGTGGCGGTGGTAGCGGTGGTGGTGGTTCTGGTGGCGGTGGCTCTGATATTGAACTGACCCAGAGCCCGGCGACCCTGAGCGTGACCCCGGGCGATAGCGTGAGCCTGAGCTGCCGCGCGAGCCAGAGCATTAGCAACAACCTGCATTGGTATCAGCAGAAAAGCCATGAACCGCCGCGCCTGCTGATTAAATATGTGAGCCAGAGCGTGAGCGGCATTCCGAGCCGCTTTAGCGGCAGCGGCAGCGGCACCGATTTTACCCTGAGCATTAACAGCGTGGAAACCGAAGATTTTGGCATGTATTTTTGCGGCCAGAGCAACAGCTGGCCGCGCACCTTTGGCGGCGGCACCAAACTGGAAATTAAACGCCTCGAGGGCGGATCCGAACAAAAGCTTATTTCTGAAGAGGACTTGTAATAG

>D44.1 (RH2.2)

GACATCGAACTCACTCAATCTCCAGCGACCCTCAGCGTTACCCCTGGTGACTCTGTGTCTCTGTCTTGCCGTGCGTCTCAGTCTATTTCTAACAATCTGCATTGGTATCAGCAAAAAAGCCATGAATCCCCTCGTCTGCTGATCAAATACGTTTCCCAATCTTCTTCTGGTATCCCATCTCGTTTCTCCGGCTCCGGCAGCGGCACTGACTTTACCCTGTCTATCAACTCCGTCGAGACGGAAGACTTCGGTATGTACTTCTGCCAGCAGAGCAACTCTTGGCCGCGTACCTTTGGCGGCGGTACCAAACTGGAAATCAAACGTAAACGAACTGTGGCTGCACCATCTGTCTTCATCTTCCCGCCATCTGATTCACAGTTGAAATCTGGAACTGCCTCTGTTGTGTGCCTGCTGAATAACTTCTATCCCAGAGAGGCCAAAGTACAGTGGAAGGTGGATAACGCCCTCCAATCGGGTAACTCCCAGGAGAGTGTCACAGAGCAGGACAGCAAGGACAGCACCTACAGCCTCAGCAGCACCCTGACGCTGAGCAAAGCAGACTACGAAAAACATAAAGTCTACGCCTGCGAAGTCACCCATCAGGGCCTGAGCTCGCCCGTCACAAAGAGCTTCAACAGGGGAGAGTGTGGTGGTTCTGATTACAAAGATGACGATGACAAATAATTAACTCGAGGCTGAGCAAAGCAGACTACTAATAACATAAAGTCTACGCCGGACGCATCGTGGCCCTAGTACGCAAGTTCACGTAAAAAGGGTAACTAGAGGTTGAGGTGATTTTATGAAAAAGAATATCGCATTTCTTCTTGCATCTATGTTCGTTTTTTCTATTGCTACAAACGCGTACGCTGAGATCTCCCAGGTTCAGCTGCAGGAATCTGGTGCGGAAGTTATGAAGCCGGGTGCGAGCGTGAAGATCTCCTGCAAAGCAACTGGTTACACCTTCTCCACCTATTGGATCGAATGGGTTAAACAACGTCCGGGTCACGGTCTGGAATGGATCGGTGAAATCCTGCCGGGCTCTGGTAGCACCTACTACAACGAAAAATTCAAAGGCAAAGCGACCTTCACCGCAGACACCTCCTCTAACACCGCGTATATGCAGCTCTCTTCTCTGACCTCTGAAGACAGCGCCGTTTACTACTGCGCGCGTGGCGACGGTAACTACGGTTACTGGGGCCAGGGTACCACCCTCACCGTTTCTTCCGCGTCCACCAAGGGTCCATCGGTCTTCCCCCTGGCACCCTCCTCCAAGAGCACCTCTGGGGGCACAGCGGCCCTGGGCTGCCTGGTCAAGGACTACTTCCCCGAACCGGTGACGGTGTCGTGGAACTCAGGCGCCCTGACCAGCGGCGTGCACACCTTCCCGGCTGTCCTACAGTCCTCAGGACTCTACTCCCTCAGCAGCGTGGTGACCGTGCCCTCCAGCAGCTTGGGCACCCAGACCTACATCTGCAACGTGAATCACAAGCCCAGCAACACCAAGGTCGACAAGAAAGTTGAGCCCAAATCTTGTGACAAAACTCACACATCTAGACACCACCACCACCACCACTAA

>D44.1^des^ (RH2.2)

GATATTGAACTGACCCAGAGCCCGGCGACCCTGAGCGTGACCCCGGGCGATAGCGTGAGCCTGAGCTGCCGCGCGAGCCAGAGCATTAGCAACAACCTGCATTGGTATCAGCAGAAAAGCCATGAACCGCCGCGCCTGCTGATTAAATATGTGAGCCAGAGCGTGAGCGGCATTCCGAGCCGCTTTAGCGGCAGCGGCAGCGGCACCGATTTTACCCTGAGCATTAACAGCGTGGAAACCGAAGATTTTGGCATGTATTTTTGCGGCCAGAGCAACAGCTGGCCGCGCACCTTTGGCGGCGGCACCAAACTGGAAATTAAACGCAAACGAACTGTGGCTGCACCATCTGTCTTCATCTTCCCGCCATCTGATTCACAGTTGAAATCTGGAACTGCCTCTGTTGTGTGCCTGCTGAATAACTTCTATCCCAGAGAGGCCAAAGTACAGTGGAAGGTGGATAACGCCCTCCAATCGGGTAACTCCCAGGAGAGTGTCACAGAGCAGGACAGCAAGGACAGCACCTACAGCCTCAGCAGCACCCTGACGCTGAGCAAAGCAGACTACGAAAAACATAAAGTCTACGCCTGCGAAGTCACCCATCAGGGCCTGAGCTCGCCCGTCACAAAGAGCTTCAACAGGGGAGAGTGTGGTGGTTCTGATTACAAAGATGACGATGACAAATAATTAACTCGAGGCTGAGCAAAGCAGACTACTAATAACATAAAGTCTACGCCGGACGCATCGTGGCCCTAGTACGCAAGTTCACGTAAAAAGGGTAACTAGAGGTTGAGGTGATTTTATGAAAAAGAATATCGCATTTCTTCTTGCATCTATGTTCGTTTTTTCTATTGCTACAAACGCGTACGCTGAGATCTCCCAGGTTCAGCTGCAGGAAAGCGGCGCGGAAGTGATGAAACCGGGCGCGAGCGTGAAAATTAGCTGCAAAGCGACCGGCTATACCTTTAGCACCTATTGGATTGAATGGATGAAACAGCGCCCGGGCGGCGGCCTGGAATATATTGGCGAAATTCTGCCGGGCAGCGGCAGCACCTATTATAACGAAAAATTTAAAGGCAAAGCGACCTTTACCGCGGATACCAGCAGCAACACCGCGTATATGCAGCTGAGCAGCCTGACCAGCGAAGATAGCGCGGTGTATTATTGCGCGCGCGGCGATGGCTATTATAAATATTGGGGCCAGGGCACCACCCTGACCGTGTCTTCCGCGTCCACCAAGGGTCCATCGGTCTTCCCCCTGGCACCCTCCTCCAAGAGCACCTCTGGGGGCACAGCGGCCCTGGGCTGCCTGGTCAAGGACTACTTCCCCGAACCGGTGACGGTGTCGTGGAACTCAGGCGCCCTGACCAGCGGCGTGCACACCTTCCCGGCTGTCCTACAGTCCTCAGGACTCTACTCCCTCAGCAGCGTGGTGACCGTGCCCTCCAGCAGCTTGGGCACCCAGACCTACATCTGCAACGTGAATCACAAGCCCAGCAACACCAAGGTCGACAAGAAAGTTGAGCCCAAATCTTGTGACAAAACTCACACATCTAGACACCACCACCACCACCACTAA

>G6 (pCTcon2)

GAAGTGCAGCTGGTGGAAAGCGGCGGCGGCCTGGTGCAGCCGGGCGGCAGCCTGCGCCTGAGCTGCGCGGCGAGCGGCTTTACCATTAGCGATTATTGGATTCATTGGGTGCGCCAGGCGCCGGGCAAAGGCCTGGAATGGGTGGCGGGCATTACCCCGGCGGGCGGCTATACCTATTATGCGGATAGCGTGAAAGGCCGCTTTACCATTAGCGCGGATACCAGCAAAAACACCGCGTATCTGCAGATGAACAGCCTGCGCGCGGAAGATACCGCGGTGTATTATTGCGCGCGCTTTGTGTTTTTTCTGCCGTATGCGATGGATTATTGGGGCCAGGGCACCCTGGTGACCGTGAGCTCTTCCGCGTCCGGTGGCGGTGGTAGCGGTGGTGGTGGTTCTGGTGGCGGTGGCTCTGATATTCAGATGACCCAGAGCCCGAGCAGCCTGAGCGCGAGCGTGGGCGATCGCGTGACCATTACCTGCCGCGCGAGCCAGGATGTGAGCACCGCGGTGGCGTGGTATCAGCAGAAACCGGGCAAAGCGCCGAAACTGCTGATTTATAGCGCGAGCTTTCTGTATAGCGGCGTGCCGAGCCGCTTTAGCGGCAGCGGCAGCGGCACCGATTTTACCCTGACCATTAGCAGCCTGCAGCCGGAAGATTTTGCGACCTATTATTGCCAGCAGAGCTATACCACCCCGCCGACCTTTGGCCAGGGCACCAAAGTGGAAATTAAACGCCTCGAGGGCGGATCCGAACAAAAGCTTATTTCTGAAGAGGACTTGTAATAG

>G6^des1^ (pCTcon2)

GAAGTGCAGCTGGTGGAAAGCGGCGGCGGCCTGGTGCAGCCGGGCGGCAGCCTGCGCCTGAGCTGCGCGGCGAGCGGCTTTACCATTAGCGATTATTGGATTCATTGGGTGCGCCAGGCGCCGGGCAAAGGCCTGGAATGGGTGGCGGGCATTACCCCGGCGGGCGGCTATACCCATTATGCGGATAGCGTGAAAGGCCGCTTTACCATTAGCGCGGATACCAGCAAAAACACCGCGTATCTGCAGATGAACAGCCTGCGCGCGGAAGATACCGCGGTGTATTTTTGCGCGCGCTTTGTGTTTTTTCTGCCGTATGCGATGGATTATTGGGGCCAGGGCACCCTGGTGACCGTGAGCTCTTCCGCGTCCGGTGGCGGTGGTAGCGGTGGTGGTGGTTCTGGTGGCGGTGGCTCTGATATTCAGATGACCCAGAGCCCGAGCAGCCTGAGCGCGAGCGTGGGCGATCGCGTGACCATTACCTGCCGCGCGAGCCAGGATGTGAGCACCGCGGTGGCGTGGTATCAGCAGAAACCGGGCAAACCGCCGAAACTGCTGATTTTTAGCGCGAGCTTTCTGTTTAGCGGCGTGCCGAGCCGCTTTAGCGGCAGCGGCAGCGGCACCGATTTTACCCTGACCATTAGCAGCCTGCAGCCGGAAGATTTTGCGACCTATTATTGCCTGCAGAGCTATACCGTGACCCCGACCTTTGGCCCGGGCACCAAAGTGGAAATTAAACGCCTCGAGGGCGGATCCGAACAAAAGCTTATTTCTGAAGAGGACTTGTAATAG

>G6^des13^ (pCTcon2)

GAAGTGCAGCTGGTGGAAAGCGGCGGCGGCCTGGTGCAGCCGGGCGGCAGCCTGCGCCTGAGCTGCGCGGCGAGCGGCTTTACCATTAGCGATTATTGGATTCATTGGGTGCGCCAGGCGCCGGGCAAAGGCCTGGAATGGGTGGCGGGCATTACCCCGGCGGGCGGCTATACCCATTATGCGGATAGCGTGAAAGGCCGCTTTACCATTAGCGCGGATACCAGCAAAAACACCGCGTATCTGCAGATGAACAGCCTGCGCGCGGAAGATACCGCGGTGTATTTTTGCGCGCGCTTTGTGTTTTTTCTGCCGTATGCGATGGATTATTGGGGCCAGGGCACCCTGGTGACCGTGAGCTCTTCCGCGTCCGGTGGCGGTGGTAGCGGTGGTGGTGGTTCTGGTGGCGGTGGCTCTGATATTCAGATGACCCAGAGCCCGAGCAGCCTGAGCGCGAGCGTGGGCGATCGCGTGACCATTACCTGCCGCGCGAGCCAGGATGTGAGCACCGCGGTGGCGTGGTATCAGCAGAAACCGGGCAAACCGCCGAAACTGCTGATTTATAGCGCGAGCTTTCTGTATAGCGGCGTGCCGAGCCGCTTTAGCGGCAGCGGCAGCGGCACCGATTTTACCCTGACCATTAGCAGCCTGCAGCCGGAAGATTTTGCGACCTATTATTGCCTGCAGAGCTATACCGATACCCCGACCTTTGGCCAGGGCACCAAAGTGGAAATTAAACGCCTCGAGGGCGGATCCGAACAAAAGCTTATTTCTGAAGAGGACTTGTAATAG
