# Supplementary material for: Optimizing antibody affinity and stability by the automated design of the variable light-heavy chain interfaces
Source: PLoS Comput Biol. 2019 Aug 23;15(8):e1007207. doi: 10.1371/journal.pcbi.1007207 (PMC6728052; doi:10.1371/journal.pcbi.1007207)
Supplement: S2 Text — Protein sequences used in the mass spectrometry analyses. (DOCX) [file pcbi.1007207.s015.docx]

**Protein sequences of G6 and G6^des13^ IgGs (used in Mass Spectrometry analysis)**

>G6 (light)

DIQMTQSPSSLSASVGDRVTITCRASQDVSTAVAWYQQKPGKAPKLLIYSASFLYSGVPSRFSGSGSGTDFTLTISSLQPEDFATYYCQQSYTTPPTFGQGTKVEIK*RT*VAAPSVFIFPPSDEQLKSGTASVVCLLNNFYPREAKVQWKVDNALQSGNSQESVTEQDSKDSTYSLSSTLTLSKADYEKHKVYACEVTHQGLSSPVTKSFNRGEC

>G6 (heavy)

EVQLVESGGGLVQPGGSLRLSCAASGFTISDYWIHWVRQAPGKGLEWVAGITPAGGYTYYADSVKGRFTISADTSKNTAYLQMNSLRAEDTAVYYCARFVFFLPYAMDYWGQGTLVTVSSA*ST*KGPSVFPLAPSSKSTSGGTAALGCLVKDYFPEXVTXSWNSGALTSGVHTFPAVLQSSGLYSLSSVVTVPSSSLGTQTYICNVNHKPSNTKVDKRVEPKSCDKTHTCPPCPAPELLGGPSVFLFPPKPKDTLMISRTPEVTCVVVDVSHEDPEVKFNWYVDGVEVHNAKTKPREEQYNSTYRVVSVLTVLHQDWLNGKEYKCKVSNKALPAPIEKTISKAKGQPREPQVYTLPPSRDEXTKNQVSLTCLVKGFYPSDIAVEWESNGQPENNYKTTPPVLDSDGSFFLYSKLTVDKSRWQQGNVFSCSVMHEALHNHYTQKSLSLSPGK

>G6^des13^ (light)

DIQMTQSPSSLSASVGDRVTITCRASQDVSTAVAWYQQKPGKPPKLLIYSASFLYSGVPSRFSGSGSGTDFTLTISSLQPEDFATYYCLQSYTDTPTFGQGTKVEIKRTVAAPSVFIFPPSDEQLKSGTASVVCLLNNFYPREAKVQWKVDNALQSGNSQESVTEQDSKDSTYSLSSTLTLSKADYEKHKVYACEVTHQGLSSPVTKSFNRGEC

>G6^des13^ (heavy)

EVQLVESGGGLVQPGGSLRLSCAASGFTISDYWIHWVRQAPGKGLEWVAGITPAGGYTHYADSVKGRFTISADTSKNTAYLQMNSLRAEDTAVYFCARFVFFLPYAMDYWGQGTLVTVSSA*ST*KGPSVFPLAPSSKSTSGGTAALGCLVKDYFPEXVTXSWNSGALTSGVHTFPAVLQSSGLYSLSSVVTVPSSSLGTQTYICNVNHKPSNTKVDKRVEPKSCDKTHTCPPCPAPELLGGPSVFLFPPKPKDTLMISRTPEVTCVVVDVSHEDPEVKFNWYVDGVEVHNAKTKPREEQYNSTYRVVSVLTVLHQDWLNGKEYKCKVSNKALPAPIEKTISKAKGQPREPQVYTLPPSRDEXTKNQVSLTCLVKGFYPSDIAVEWESNGQPENNYKTTPPVLDSDGSFFLYSKLTVDKSRWQQGNVFSCSVMHEALHNHYTQKSLSLSPGK
